# Novel Energetic Coordination Polymers Based on 1,5-Di(nitramino)tetrazole With High Oxygen Content and Outstanding Properties: Syntheses, Crystal Structures, and Detonation Properties

**DOI:** 10.3389/fchem.2019.00672

**Published:** 2019-10-15

**Authors:** Yanan Li, Tao Yu, Yiying Zhang, Jianjian Hu, Tao Chen, Yinglei Wang, Kangzhen Xu

**Affiliations:** ^1^State Key Laboratory of Fluorine & Nitrogen Chemicals, Xi'an Modern Chemistry Research Institute, Xi'an, China; ^2^School of Chemistry and Chemical Engineering, Southeast University, Nanjing, China; ^3^School of Chemical Engineering, Northwest University, Xi'an, China

**Keywords:** primary explosive, bimetallic energetic coordination, synthesis, crystal structure, property

## Abstract

In this study, a series of novel 1,5-di(nitramino)tetrazole (DNAT)-based bimetallic energetic coordination polymers, MK_2_(DNAT)_2_·4H_2_O [M = Fe, Cu, Ni, Co, and Zn], were designed and synthesized in a simple and convenient self-assembly synthetic process. The obtained compounds were fully characterized by IR spectroscopy, multinuclear NMR spectroscopy, elemental analysis, and differential scanning calorimetry (DSC). Additionally, the structures of target compounds were confirmed by single-crystal X-ray diffraction. Based on the room-temperature X-ray densities (2.095–2.138 g cm^−3^) and the calculated (CBS–QB3) heats of formation (−41.3 to 170.5 kJ mol^−1^), the detonation properties such as detonation velocities (8,147.0–8,478.4 m s^−1^) and detonation pressures (29.7–32.8 GPa) were computed using the EXPLO5 v6.04 program. Their excellent energetic properties indicated that they could serve as promising “green” primary explosives for replacement of lead azide (LA).

## Introduction

**Scheme 1:**
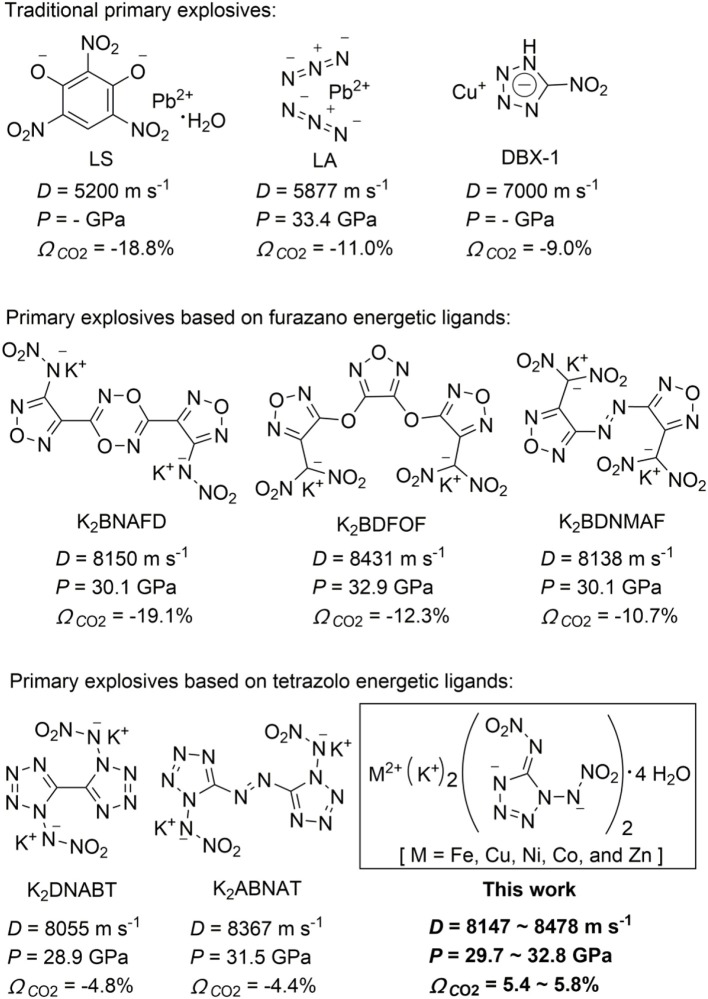
Structures and properties of several kinds of primary explosives.

In military and commercial blasting, metal-based explosives have been designed and synthesized as primary explosives [e.g., lead azide (LA)]. Primary explosives as a kind of special explosives show high sensitivity toward impact, friction, shock, heat, and electrostatic discharge and can generate an extremely rapid deflagration-to-detonation transition (Matyas and Pachman, 2013; Mehta et al., 2014; Zhang and Shreeve, 2014), which are widely used in detonators, primers, blasting caps, and initiators. Primary explosives that can generate a shockwave to transfer are used to initiate larger masses of secondary explosives such as RDX (1,3,5-trinitro-1,3,5-triazacyclohexane), HMX (1,3,5,7-tetranitro-1,3,5,7-tetraazacyclooctane), and others (He and Shreeve, 2016; Chen et al., 2018). Metal-based explosives have been designed and synthesized as primary explosives because of their high impact and friction sensitivities (Klapötke and Mehta, 2014; Shen et al., 2017). To date, LA is the most widely used primary explosive, and other traditional primary explosives include lead styphnate monohydrate (LS) (Ilyushin et al., 2012; Klapötke, 2012) and lead-free primary explosive copper(I) nitrotetrazolate (DBX-1) (Scheme 1) (Klapötke et al., 2013). However, lead contamination in air and soil has attracted people's attention in recent years, which poses a significant threat to personal safety and environmental pollution (Ilyushin et al., 2012; Klapötke, 2012) Furthermore, lead is very difficult to remove once it has been dissolved in the human blood (Matyas and Pachman, 2013). Based on environmental regulations and human health problems, there is a need to develop “green” replacements of lead-based primary explosives (Huynh et al., 2006). “Green” lead-based replacements need to consider the following criteria: the materials (a) must be safe to handle and possess a rapid deflagration to detonation transition; (b) must be thermally stable to >150°C; (c) also should possess high detonation performance and sensitivity; (d) should have long-term chemical stability; (e) should not contain toxic heavy metals or other known toxins; and (f) should be easy to synthesize and affordable (Ilyushin et al., 2012; Klapötke, 2012; Mehta et al., 2014).

Currently, energetic coordination polymers have been explored in materials science and coordination chemistry. As is well known, potassium is an environmentally friendly species with good coordinating ability to a variety of energetic ligands (Zhai et al., 2015; Li Y. N. et al., 2017). Potassium-based energetic coordination polymers are considered to be promising “green” replacements of lead-based primary explosives. Recently, several new energetic coordination polymers as promising primary explosives based on furazano and tetrazolo energetic ligands, such as potassium 3-nitramino-4-tetrazolefurazanate (K_2_NATF) (Li Y. et al., 2017), potassium 3,6-bis(4-nitramino-1,2,5-oxadiazol-3-yl)-1,2,4,5-dioxadiazinate (K_2_BNAFD) (Li Y. et al., 2017), potassium 3,4-bis(3-dinitromethylfurazan-4-oxy)furazanate (K_2_BDFOF) (Zhai et al., 2015; Li Y. et al., 2017), potassium 4,4′-bis(dinitromethyl)-3,3′-azofurazanate (K_2_BDNMAF) (Tang et al., 2016), potassium 3-dinitromethyl-4-nitraminofurazanate (K_2_DNMNAF) (Li Y. N. et al., 2017), potassium 3,4-dinitraminofurazanate (K_2_DNAF) (Li Y. et al., 2017) and potassium 4,5-bis(dinitromethyl)furoxanate (K_2_BDNMF) (He and Shreeve, 2016), potassium 1,1′-dinitramino-5,5′-bistetrazolate (K_2_DNABT) (Fischer et al., 2014; Yedukondalu and Vaitheeswaran, 2015), and potassium 1,1′-dinitramino-5,5′-azobitetrazole (K_2_ABNAT) (Fischer et al., 2016; Li Y. N. et al., 2017) were reported (Scheme 1). However, most of the above-reported coordination polymers do not own excellent detonation properties and have negative oxygen balances (OBs). Furthermore, the initiability and priming ability of the potassium-based compounds are weaker than those of LA (Chen et al., 2018) Therefore, much effort will be put in the development of other metal-based and high-energy green primary explosives that could serve as replacements for LA.

In the process of seeking green candidates for primary explosives, we focused on the use of nitrogen-rich energetic ligands, the main detonation product of which is nontoxic nitrogen gas. Meanwhile, they possess higher heats of formation, which could provide high energy output (Mehta et al., 2014). In this work, we focus our attentions on bimetallic energetic coordination polymers with high positive OB and outstanding energetic properties. A new high energetic ligand with a tetrazole ring and nitroamino groups, namely, 1,5-di(nitramino)tetrazole (DNAT), was used to successfully prepare novel high-energy-density compounds. DNAT has great detonation properties (*D* = 9,967 m s^−1^, and *P* = 43.4 GPa) (Fischer et al., 2015). Herein, we report the design and syntheses of a series of DNAT-based bimetallic energetic coordination polymers, MK_2_(DNAT)_2_·4H_2_O [M = Fe (**1**), Cu (**2**), Ni (**3**), Co (**4**), and Zn (**5**)], all of which contain one potassium and another lead-free metal atom to regulate performances.

## Syntheses

**Scheme 2:**
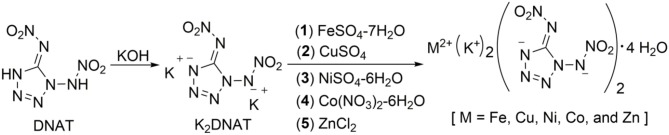
Syntheses of 1,5-di(nitramino)tetrazole-based bimetallic energetic coordination polymers.

In our study, the target compounds, DNAT-based bimetallic energetic coordination polymers, MK_2_(DNAT)_2_·4H_2_O [M = Fe (**1**), Cu (**2**), Ni (**3**), Co (**4**), and Zn (**5**)], are prepared from potassium 1,5-di(nitramino)tetrazolate (K_2_DNAT) in a self-assembly process, and the synthetic pathway is described and shown in Scheme 2. Compound DNAT is reacted with potassium hydroxide to yield K_2_DNAT. The bimetallic energetic coordination polymers, MK_2_(DNAT)_2_·4H_2_O [M = Fe, Cu, Ni, Co, and Zn], could be obtained via metathesis reactions of K_2_DNAT with FeSO_4_·7H_2_O, CuSO_4_, NiSO_4_·6H_2_O, Co(NO_3_)_2_·6H_2_O, and ZnCl_2_, respectively. The target compounds **1**–**5** could be isolated by filtering and washing with methanol or ethanol. The structures of compounds **1**–**5** were confirmed by IR spectroscopy, multinuclear NMR spectroscopy, elemental analysis, and single-crystal X-ray diffraction. The colors of single crystals **1**–**5** are yellow, green, green, orange–red, and light yellow, respectively. All of them were insensitive to light and very stable upon storage under conditions. The compounds **1**–**5** were not soluble in general organic solvents, such as methanol, ethanol, dimethylformamide (DMF), and dimethyl sulfoxide (DMSO), but did dissolve in water.

## X-Ray Crystallography

**Figure 1:**
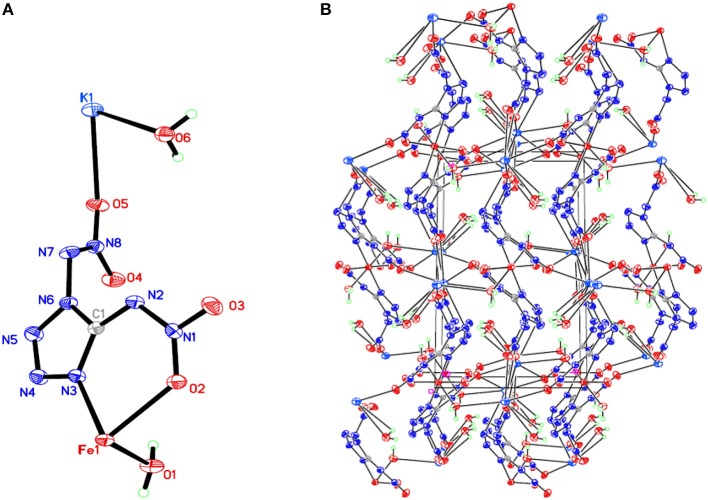
**(A)** X-ray structure of compound **1** with thermal ellipsoids at 50% probability. **(B)** Packing diagram of compound **1** viewed down the *a* axis. Symmetry codes: #1 –*x* + 1, –*y*, –*z* + 2; #2 *x* – 1, *y, z*; #3 –*x* – 1, *y* – 1/2, –*z* + 3/2; #4 –*x* – 1, –*y*, –*z* + 1; #5 *x* – 1, –*y* + 1/2, *z* – 1/2; #6 –*x, y* – 1/2, –*z* + 3/2; #7 –*x* – 2, –*y*, –*z* + 1; #8 *x* + 1, *y, z*; #9 –*x, y* + 1/2, –*z* + 3/2; #10 –*x* – 1, *y* + 1/2, –*z* + 3/2; #11 *x* + 1, –*y* + 1/2, *z* + 1/2.

**Figure 2:**
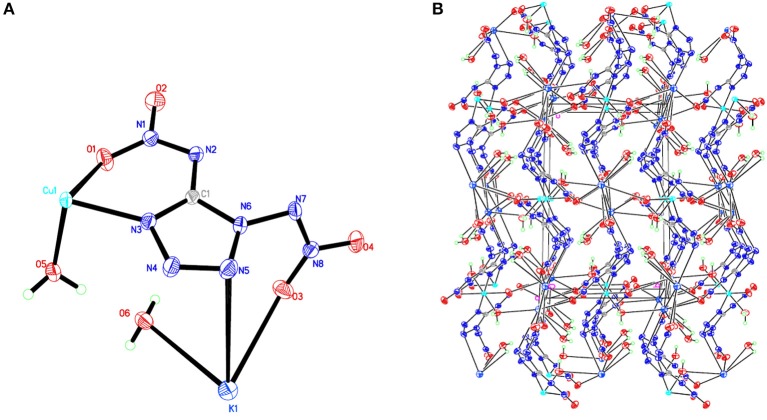
**(A)** X-ray structure of compound **2** with thermal ellipsoids at 50% probability. **(B)** Packing diagram of compound **2** viewed down the *a* axis. Symmetry codes: #1 –*x* + 1, –*y*, –*z*; #2 *x*, –*y* + 1/2, *z* – 1/2; #3 –*x* + 1, *y* – 1/2, –*z* + 1/2; #4 *x*, –*y* + 3/2, *z* + 1/2; #5 *x*, –*y* + 1/2, *z* + 1/2; #6 –*x, y* + 1/2, –*z* + 1/2; #7 –*x* + 1, *y* + 1/2, –*z* + 1/2; #8 *x*, –*y* + 3/2, *z* – 1/2; #9 –*x, y* – 1/2, –*z* + 1/2.

**Figure 3:**
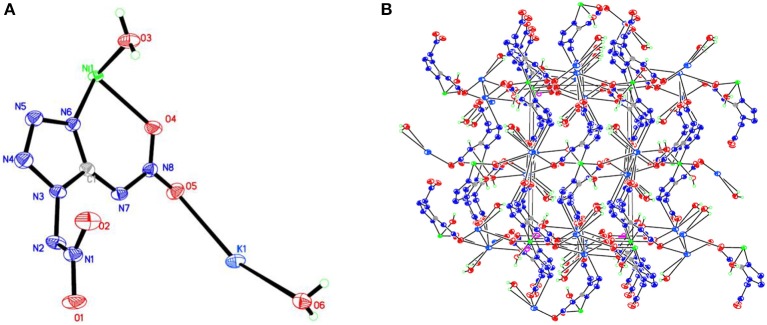
**(A)** X-ray structure of compound **3** with thermal ellipsoids at 50% probability. **(B)** Packing diagram of compound **3** viewed down the *a* axis. Symmetry codes: #1 –*x* + 3, *y* + 1/2, –*z* + 1/2; #2 –*x* + 2, *y* + 1/2, –*z* + 1/2; #3 *x*, –*y* + 1/2, *z* – 1/2; #4 –*x* + 2, –*y* + 1, –*z*; #5 *x* + 1, *y, z*; #6 –*x* + 1, –*y*, –*z*; #7 *x*, –*y* + 1/2, *z* + 1/2; #8 –*x* + 2, *y* – 1/2, –*z* + 1/2; #9 –*x* + 3, *y* – 1/2, –*z* + 1/2; #10 *x* – 1, *y, z*.

**Figure 4:**
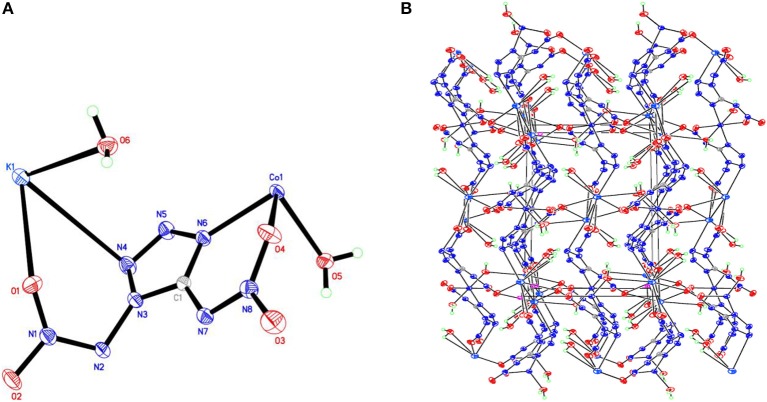
**(A)** X-ray structure of compound **4** with thermal ellipsoids at 50% probability. **(B)** Packing diagram of compound **4** viewed down the *a* axis. Symmetry codes: #1 *x* – 1, *y, z*; #2 –*x* + 1, *y* – 1/2, –*z* + 1/2; #3 –*x* + 1, –*y* + 1, –*z* + 1; #4 *x*, –*y* + 3/2, *z* + 1/2; #5 *x*, –*y* + 1/2, *z* + 1/2; #6 –*x*, –*y* + 1, –*z* + 1; #7 –*x*, –*y* + 1, –*z*; #8 *x* + 1, *y, z*; #9 –*x* + 1, *y* + 1/2, –*z* + 1/2; #10 *x*, –*y* + 3/2, *z* – 1/2; #11 *x*, –*y* + 1/2, *z* – 1/2.

**Figure 5:**
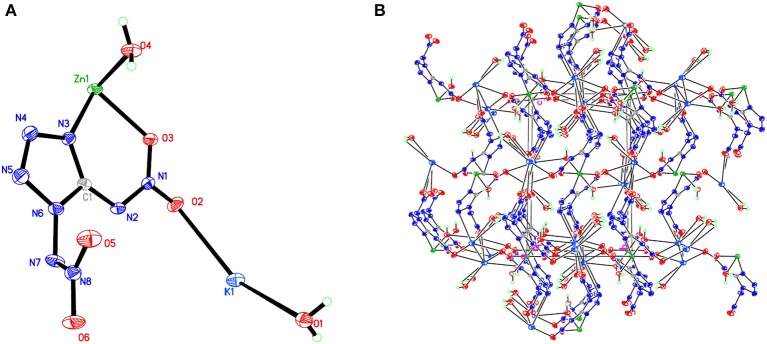
**(A)** X-ray structure of compound **5** with thermal ellipsoids at 50% probability. **(B)** Packing diagram of compound **5** viewed down the *a* axis. Symmetry codes: #1 –*x*, –*y*, –*z*; #2 –*x* + 2, *y* + 1/2, –*z* + 1/2; #3 –*x* + 1, *y* + 1/2, –*z* + 1/2; #4 *x*, –*y* + 1/2, *z* – 1/2; #5 *x* + 1, *y, z*; #6 –*x* + 1, –*y* + 1, –*z*; #7 –*x* + 2, –*y* + 1, –*z*; #8 –*x* + 1, *y* – 1/2, –*z* + 1/2; #9 *x*, –*y* + 1/2, *z* + 1/2; #10 *x* – 1, *y, z*; #11 –*x* + 2, *y* – 1/2, –*z* + 1/2.

Room-temperature (296 K) X-ray diffraction was used to determine all of the target compounds **1**–**5** and to obtain accurate densities for property calculations. Suitable single crystals of compounds **1**–**5** for X-ray diffraction measurements were grown by slow evaporation from a water solution. The X-ray structures and packing diagrams of single crystals for compounds **1**–**5** are shown in Figures 1–5, and the crystallographic data for compounds **1**–**5** are summarized in Tables 1, 2.

**Table 1:** Crystallographic data for compounds **1**–**3**.

**Compounds**	**1**	**2**	**3**
CCDC	1,826,491	1,826,485	1,826,494
Empirical formula	C_2_H_8_N_16_O_12_FeK_2_	C_2_H_8_N_16_O_12_CuK_2_	C_2_H_8_N_16_O_12_NiK_2_
Formula weight (g mol^−1^)	581.91	588.91	583.91
Temperature (K)	296	296	296
Crystal system	Monoclinic	Monoclinic	Monoclinic
Space group	*P*2(1)/*c*	*P*2(1)/*c*	*P*2(1)/*c*
Unit cell dimensions	*a* = 7.3905(7) Å, α = 90°	*a* = 7.3926(9) Å, α = 90°	*a* = 7.405(2) Å, α = 90°
	*b* = 8.8773(9) Å, β = 103.726(2)°	*b* = 8.9423(12) Å, β = 104.458(2)°	*b* = 8.940(3) Å, β = 104.524(4)°
	*c* = 14.4845(14) Å, γ = 90°	*c* = 14.3368(18) Å, γ = 90°	*c* = 14.365(4) Å, γ = 90°
Volume (Å^3^)	923.16 (16)	917.7 (2)	920.6 (5)
Formula *Z*	2	1	2
Calculated density (g cm^−3^)	2.095	2.135	2.111
Absorption correction	Multi-scan	Multi-scan	Multi-scan
Theta range for data collection (°)	2.71–25.10	2.71–27.12	2.71–25.09
Refinement method	Full-matrix least squares on *F*^2^	Full-matrix least squares on *F*^2^	Full-matrix least squares on *F*^2^
Goodness of fit on *F*^2^	1.072	1.062	1.038
Final *R* indices[*I* > 2sigma(*I*)]	*R*_1_ = 0.0207, *wR*_2_ = 0.0559	*R*_1_ = 0.0500, *wR*_2_ = 0.1144	*R*_1_ = 0.0355, *wR*_2_ = 0.0939
*R* indices (all data)	*R*_1_ = 0.0223, *wR*_2_ = 0.0569	*R*_1_ = 0.0611, *wR*_2_ = 0.1221	*R*_1_ = 0.0410, *wR*_2_ = 0.0989
Largest diff. peak and hole (e·Å^−3^)	0.256 and −0.224	1.848 and −1.859	0.552 and −0.544
*R*_1_ = Σ(*F*_o_ – *F*_c_)/Σ*F*_o_, *wR*_2_ = [Σ*w*($F_{\text{o}}^{2}$ – $F_{\text{c}}^{2}$)^2^/Σ*w*($F_{\text{o}}^{2}$)^2^]^1/2^			

*CCDC, Cambridge Crystallographic Data Center*.

**Table 2:** Crystallographic data for compounds **4** and **5**.

**Compounds**	**4**	**5**
CCDC	1,826,488	1,848,490
Empirical formula	C_2_H_8_N_16_O_12_CoK_2_	C_2_H_8_N_16_O_12_ZnK_2_
Formula weight (g mol^−1^)	584.91	589.91
Temperature (K)	296	296
Crystal system	Monoclinic	Monoclinic
Space group	*P*2(1)/*c*	*P*2(1)/*c*
Unit cell dimensions	*a* = 7.3649(9) Å, α = 90°	*a* = 7.4044(10) Å, α = 90°
	*b* = 8.8847(12) Å, β = 104.004(2)°	*b* = 8.8825(12) Å, β = 104.281(2)°
	*c* = 14.4193(18) Å, γ = 90°	*c* = 14.4234(19) Å, γ = 90°
Volume (Å^3^)	915.5 (2)	919.3 (2)
Formula *Z*	2	2
Calculated density (g cm^−3^)	2.124	2.138
Absorption correction	Multi-scan	Multi-scan
Theta range for data collection (°)	2.72–26.24	2.72–25.09
Refinement method	Full-matrix least squares on *F*^2^	Full-matrix least squares on *F*^2^
Goodness of fit on *F*^2^	0.971	1.064
Final *R* indices[*I* > 2sigma(*I*)]	*R*_1_ = 0.0246, *wR*_2_ = 0.0717	*R*_1_ = 0.0254, *wR*_2_ = 0.0614
*R* indices (all data)	*R*_1_ = 0.0295, *wR*_2_ = 0.0892	*R*_1_ = 0.0307, *wR*_2_ = 0.0642
Largest diff. peak and hole (e·Å^−3^)	0.264 and −0.368	0.280 and −0.368
*R*_1_ = Σ(*F*_o_ – *F*_c_)/Σ*F*_o_, *wR*_2_ = [Σ*w*($F_{\text{o}}^{2}$ – $F_{\text{c}}^{2}$)^2^/Σ*w*($F_{\text{o}}^{2}$)^2^]^1/2^		

*CCDC, Cambridge Crystallographic Data Center*.

Compound **1**, which crystallizes as yellow prism in the monoclinic space group *P*2(1)/*c* with a density of 2.095 g cm^−3^ and a cell volume of 923.2 Å^3^, contains two molecules of water and consists of two molecules per unit cell. Compound **2** crystallizes in the monoclinic space group *P*2(1)/*c* with a density of 2.250 g cm^−3^ and a cell volume of 817.7 Å^3^. The repeating unit of compound **2** contains two potassium ions, one cupric ion and two K_2_DNAT anions. Compound **3** crystallizes in the monoclinic space group *P*2(1)/*c* with two formulas per unit cell, and it possesses a calculated density of 2.111 g cm^−3^. The unit cell of compound **4**, which crystallizes in the monoclinic space group *P*2(1)/*c* with a density of 2.124 g cm^−3^, contains two formula moieties, whereas compound **5** also crystallizes in the monoclinic space group *P*2(1)/*c* with two molecules per unit cell and has a density of 2.138 g cm^−3^. For all target compounds **1**–**5**, because of the average tendency of electronic and chemical bond lengths of the conjugated system in K_2_DNAT anions, the C–N bond lengths are in the range of 1.325–1.366 Å, shorter than that of the normal C–N single bond (1.480 Å) and longer than that of the normal C=N double bond (1.270 Å). The N–N bond lengths lie between 1.274 and 1.396 Å, which are shorter than the lengths of normal N–N single bond (1.460 Å) and longer than N=N double bond (1.250 Å) (Allen et al., 1987; Tang et al., 2013). The nitrogen atoms N2 and N7 and all the atoms in the tetrazole ring are nearly coplanar except the N–nitramino groups. The nitramino moieties attached to the carbon are almost planar with the tetrazole ring (≮O(2)–N(1)–N(2)–C(1) 1.8(2)°, ≮O(1)–N(1)–N(2)–C(1) 7.7(3)°, ≮C(1)–N(7)–N(8)–O(4) 3.4(4)°, ≮C(1)–N(7)–N(8)–O(4) 0.8(3)°, and ≮O(3)–N(1)–N(2)–C(1) 2.4(3)° for compounds **1**–**5**, respectively), and all the *N*-nitramino groups are twisted out of this plane with the dihedral angle of −74.83(19)°, 95.1(2)°, −77.3(3)°, −76.1(2)°, and −76.0(2)° for target compounds **1**–**5**, respectively. The K ions in all single crystals of the compounds **1**–**5** are irregularly eight-coordinated pattern by six oxygen atoms from nitramino groups and water molecules and two nitrogen atoms from tetrazolo rings and nitramino groups, whereas the divalent metal ions adopt a six-coordinated pattern by four oxygen atoms from nitramino groups and water molecules and two nitrogen atoms, all from tetrazolo rings. As a result, the dimetallic cations connecting closely with K_2_DNAT dianionic ligands and water molecules generate 3D network crystal packing structures. More detailed information about crystallographic date collection and structure refinement can be found in the Supplementary Material.

## Physicochemical and Energetic Properties

Thermal stabilities of all energetic compounds **1**–**5** were studied with differential scanning calorimetry (DSC) and thermogravimetry (TG) at a linear heating rate of 5°C min^−1^. In the TG and DSC curves (Supplementary Material, Figures S6–S10), compounds **1**–**5** started to decompose at 153.8°C, 167.1°C, 208.9°C, 185.4°C, and 178.0°C (onset temperatures) without melting, respectively. There are no endothermic peaks for compounds **1**–**5** that indicate that target compounds melt with concomitant decomposition under the heating conditions. The thermal decomposition temperatures of compounds **1**–**5** are higher than the criterion of 150°C for “green” primary explosives (Mehta et al., 2014). For all the compounds **1**–**5**, other mass losses are observed between 88.0°C and 134.7°C, corresponding to the loss of four coordinated water molecules. Furthermore, a rapid and strong explosion occurs at onset temperature for each compound. Notably, these energetic coordination polymers have been stored at room temperatures (25~35°C) for more than 1 year, and their weights and colors have not changed, which illustrates that they hold good stability for a long time at room temperatures.

The mechanical sensitivities toward impact and friction were determined experimentally according to BAM methods by using a standard BAM Fallhammer and a BAM Friction tester^1^^,^^2^ (United Nations Publication, 2009). The impact and friction sensitivities of the target compounds **1**–**5** are between 3 and 6 J and 16 and 26 N, respectively. The impact sensitivities are similar with those of LA. Therefore, they should be considered as primary explosives to be handled with appropriate precautions (Ilyushin et al., 2012; Klapötke, 2012; Mehta et al., 2014).

OB is an important characteristic for energetic materials. All of target compounds **1**–**5** have positive OBs in the range of 5.4% to 5.8% (based on CO_2_), which are much higher than those of traditional primary explosives LA (−11.0%) and other reported potassium-based primary explosives based on furazano or tetrazolo energetic ligands (−23.4 to 4.3%, shown in Scheme 1). The OB results indicated that the chemical energy of the compounds could be fully utilized when the detonation reactions were happening, which could improve the detonation properties of energetic materials. In addition, all of the compounds **1**–**5** possess a higher content of nitrogen and oxygen, which is more than 70.0% (shown in Table 3).

**Table 3:** Properties of DNAT-based bimetallic energetic coordination polymers and Pb(N_3_)_2_.

**Compounds**	**1**	**2**	**3**	**4**	**5**	**Pb(N_**3**_)_**2**_ (Zhai et al., 2015; Li Y. et al., 2017)**
Formula	C_2_H_8_N_16_O_12_FeK_2_	C_2_H_8_N_16_O_12_CuK_2_	C_2_H_8_N_16_O_12_NiK_2_	C_2_H_8_N_16_O_12_CoK_2_	C_2_H_8_N_16_O_12_ZnK_2_	N_6_Pb
*T*_dec_ (°C)*^a^*	153.8	167.1	208.9	185.4	178.0	315.0
Ω (CO) (%)*^b^*	11.0	10.9	11.7	10.9	10.8	−11.0
Ω (CO_2_) (%)*^c^*	5.5	5.4	5.8	5.5	5.4	−11.0
N + O (%)*^d^*	71.5	70.5	70.0	71.1	70.3	28.9
ρ (g cm^−3^)*^e^*	2.095	2.135	2.111	2.124	2.138	4.8
Δ*H*_f_ (kJ mol^−1^) *^f^*	73.2	−41.3	74.6	170.5	71.3	450.1
*D* (m s^−1^)*^g^*	8,147.0	8,348.4	8,169.1	8,187.5	8,478.4	5,877
*P* (GPa)*^h^*	29.8	31.5	29.7	30.3	32.8	33.4
*Q* (kJ kg^−1^) *^i^*	4,910.7	3,437.9	4,798.4	5,143.1	3,750.6	1,569
*T*_det_ (K) *^j^*	3,492	2,702	3,389	3,505	2,871	3,353
*V*_0_ (L kg^−1^)*^k^*	586.9	579.4	584.0	577.0	577.5	230
IS (J)*^l^*	3	3	6	5	4	2.5–4
FS (N)*^m^*	16	16	26	22	18	0.1–1

a *Temperature of thermal decomposition according to differential scanning calorimetry (DSC) (5°C min^−1^)*.

b *Oxygen balance (based on CO and K_2_O) for C_a_H_b_O_c_N_d_, 1,600(c – a – b/2)/M_W_, M_W_, molecular weight*.

c *Oxygen balance (based on CO_2_ and K_2_O) for C_a_H_b_O_c_N_d_, 1,600(c – 2a – b/2)/M_W_, M_W_, molecular weight*.

d *Nitrogen and oxygen content*.

e *Single-crystal density at 296 K*.

f *Heat of formation*.

g *Detonation velocity*.

h *Detonation pressure*.

i *Heat of detonation*.

j *Detonation temperature calculated with EXPLO5 v6.04*.

k *Gas volume after detonation calculated with EXPLO5 v6.04*.

l *Impact sensitivity*.

m *Friction sensitivity*.

*DNAT, 1,5-di(nitramino)tetrazole*.

The densities of bimetallic energetic compounds **1**–**5** were confirmed using single-crystal X-ray diffraction at 296 K, which lies in the range of 2.095 to 2.138 g cm^−3^. The heats of formation of compounds **1**–**5** were calculated to be from −41.3 to 170.5 kJ mol^−1^ by the use of the Gaussian 09 (Revision B.01) suite of programs at the level of theory of DFT with the method of CBS-QB3. In order to explore the properties of bimetallic energetic compounds, several detonation parameters of compounds **1**–**5** were calculated with the EXPLO5 code (Sucéska, 2017) in its version 6.04 based on the crystal densities and calculated heats of formation. As can be seen in Table 3, compounds **1**–**5** showed remarkable detonation values, and the calculated detonation velocities (8,147.0–8,478.4 m s^−1^) and detonation pressures (29.7–32.8 GPa) are comparable with those of traditional lead-based primary explosives such as LA (5,877.0 m s^−1^, 33.4 GPa). Noteworthy, DNAT-based bimetallic energetic compounds **1**–**5** displayed excellent overall performance as suitable and non-toxic green replacements for lead-based primary explosives.

## Conclusions

In conclusion, a series of DNAT-based bimetallic energetic coordination polymers, MK_2_(DNAT)_2_·4H_2_O [M = Fe, Cu, Ni, Co, and Zn], were first designed and synthesized via a convenient self-assembly synthetic process from K_2_DNAT, and the structures of target compounds were confirmed by single-crystal X-ray diffraction. The target compounds display high densities (2.095–2.138 g cm^−3^), good positive OBs (5.4–5.8%), acceptable thermal stabilities (onset temperature, 153.8–208.9°C), and remarkable detonation properties (*D* = 8,147.0–8,478.4 m s^−1^, *P* = 29.7–32.8 GPa), which make them easily outperform the widely used primary explosive, LA (*D* = 5,877 m s^−1^, *P* = 33.4 GPa, Ω_CO2_ = −11.0%). Noteworthy are their excellent energetic properties that make them competitive replacements as environmentally friendly primary explosives for lead(II) azide, which contains toxic ingredient.

## Materials and Methods

**Caution!** All of DNAT-based bimetallic energetic coordination polymers are potential primary explosives and may explode under certain conditions. Proper safety precautions should be taken when handling these compounds. Laboratories and personnel should be properly grounded, and safety equipment such as leather gloves, face shield, and ear plugs are recommended.

### General Information

All chemical reagents and solvents were used as supplied unless otherwise stated. Elemental analyses (C, H, and N) were performed on a VARI-El-3 elementary analysis instrument. Infrared spectra were obtained from KBr pellets on a Nicolet NEXUS 870 Infrared spectrometer in the range of 400–4,000 cm^−1^. ^13^C NMR was obtained in D_2_O-*d*_2_ on a Bruker AV 500 NMR spectrometer. The DSC experiments were performed using a DSC-Q 200 apparatus (TA, USA) under a nitrogen atmosphere at a flow rate of 50 ml min^−1^. About 0.1–0.5 mg of the samples were sealed in aluminum pans for DSC. Impact and friction sensitivity measurements were determined using a BAM drophammer and a BAM friction tester^1^,^2^ (United Nations Publication, 2009). Energetic properties have been calculated with the EXPLO5 v6.04 program (Sucéska, 2017) code using the X-ray crystal densities at room temperature and calculated solid state heats of formation.

### Theoretical Studies

Calculations were performed using the Gaussian 09 suite of programs (Frisch et al., 2009). The geometric optimization of the structures and frequency analyses employed the density functional theory (DFT) B3LYP method with PBE exchange (Perdew et al., 1996) and correlation and plane wave basis set realized by the CASTEP code (Clark et al., 2005). Each optimized structure was characterized to determine the true local energy minima on the potential energy surface without imaginary frequencies. The heats of formation (Δ*H*_f_) of the target compounds **1**–**5** was computed using the CBS-QB3 method (Montgomery et al., 1999, 2000). With the use of the room-temperature X-ray densities and calculated heats of formation, the detonation properties were calculated using the EXPLO5 v6.04 program (Sucéska, 2017) according to the Kamlet–Jacobs equations (Kamlet and Ablard, 1968; Kamlet and Dicknison, 1968; Kamlet and Jacobs, 1968).

### Experimental Procedures

General procedure for synthesis of compounds **1**–**5**: Compound K_2_DNAT (0.133 g, 0.5 mmol) was suspended in distilled water (3.0 ml) at room temperature and stirred until the solid was fully dissolved. Inorganic-metallic salts (0.75 mmol), such as FeSO_4_·7H_2_O (0.209 g), CuSO_4_ (0.120 g), NiSO_4_·6H_2_O (0.197 g), Co(NO_3_)_2_·6H_2_O (0.218 g), and ZnCl_2_ (0.102 g), were added to the reaction solution. After being stirred for 10 min, the reaction mixture was heated to 50–55°C for 1–2 h. The solvents were evaporated under high vacuum. The crystals of target compounds **1**–**5** were obtained, filtered, washed with ethanol, and dried in the air.

Compound **1**: Yellow crystals, with a yield of 30.9%. IR (KBr): υ = 3,546, 3,433, 1,528, 1,438, 1,422, 1,350, 1,290, 1,112, 1,029, 920 cm^−1^; ^13^C NMR (125 MHz, D_2_O-*d*_2_, 25°C): δ = 154.11 ppm; elemental analysis calcd for C_2_H_8_N_16_O_12_FeK_2_ (581.91 g mol^−1^): C 4.13, H 1.38, N 38.49%; found: C 4.21, H 1.47, N 38.36%.

Compound **2**: Green crystals, with a yield of 44.8%. IR (KBr): υ = 3,551, 3,430, 1,522, 1,440, 1,419, 1,352, 1,292, 1,109, 1,031, 918, 863 cm^−1^; ^13^C NMR (125 MHz, D_2_O-*d*_2_, 25°C): δ = 154.16 ppm; elemental analysis calcd for C_2_H_8_N_16_O_12_CuK_2_ (588.91 g mol^−1^): C 4.07, H 1.37, N 37.99%; found: C 4.18, H 0.31, N 37.88%.

Compound **3**: Green crystals, with a yield of 37.8%. IR (KBr): υ = 3,531, 3,421, 1,521, 1,441, 1,415, 1,359, 1,288, 1,106, 1,036, 921, 859 cm^−1^; ^13^C NMR (125 MHz, D_2_O-*d*_2_, 25°C): δ = 154.12 ppm; elemental analysis calcd for C_2_H_8_N_16_O_12_NiK_2_ (583.91 g mol^−1^): C 4.11, H 1.38, N 38.30%; found: C 4.22, H 1.25, N 38.36%.

Compound **4**: Organic-red crystals, with a yield of 49.2%. IR (KBr): υ = 3,523, 3,432, 1,523, 1,442, 1,413, 1,360, 1,287, 1,103, 1,038, 916, 861 cm^−1^; ^13^C NMR (125 MHz, D_2_O-*d*_2_, 25°C): δ = 154.85 ppm; elemental analysis calcd for C_2_H_8_N_16_O_12_CoK_2_ (584.91 g mol^−1^): C 4.10, H 1.38, N 38.29%; found: C 4.22, H 1.50, N 38.17%.

Compound **5**: Light-yellow crystals, with a yield of 41.5%. IR (KBr): υ = 3,511, 3,441, 1,530, 1,444, 1,418, 1,356, 1,291, 1,107, 1,041, 919, 859 cm^−1^; ^13^C NMR (125 MHz, D_2_O-*d*_2_, 25°C): δ = 154.23 ppm; elemental analysis calcd for C_2_H_8_N_16_O_12_ZnK_2_ (589.91 g mol^−1^): C 4.06, H 1.36, N 37.87%; found: C 4.18, H 1.27, N 37.98.

## Data Availability Statement

All datasets generated for this study are included in the manuscript/Supplementary Files.

## Author Contributions

YL designed the research and performed the syntheses of target compounds and the drafting of manuscript. TY contributed to the theoretical calculation studies of target compounds. YZ and KX performed the thermal behavior research. TC and JH were involved in compound structure characterization and data analysis. YW participated in drafting the manuscript.

### Conflict of Interest

The authors declare that the research was conducted in the absence of any commercial or financial relationships that could be construed as a potential conflict of interest.

## Footnotes

^1^13.4.2 Test 3 (ii): *BAM Fallhammer*, p. 75–82.

^2^13.5.1 Test 3 (i): *BAM Friction Apparatus*, p. 104–107.
