# Supplementary material for: Novel Energetic Coordination Polymers Based on 1,5-Di(nitramino)tetrazole With High Oxygen Content and Outstanding Properties: Syntheses, Crystal Structures, and Detonation Properties
Source: Front Chem. 2019 Oct 15;7:672. doi: 10.3389/fchem.2019.00672 (PMC6803599; doi:10.3389/fchem.2019.00672)
Supplement: Supplementary file 1 [file Presentation_1.pdf]

Supplementary Material

Novel energetic coordination polymers based on 1,5-di(nitramino)tetrazole with high oxygen content and outstanding properties: syntheses, crystal structures, and detonation properties

Yanan Li[1*], Tao Yu[1,2], Yiying Zhang[1], Jianjian Hu[1], Tao Chen[1], Yinglei Wang[1] and Kangzhen Xu[3*]

[1]State Key Laboratory of Fluorine & Nitrogen Chemicals, Xi'an Modern Chemistry Research Institute, Xi'an, 710065, China
[2]School of Chemistry and Chemical Engineering, Southeast University, Nanjing, 211189, China
[3]School of Chemical Engineering, Northwest University, Xi'an, 710069, China

*** Correspondence:**
Yanan Li
lyn2003080094@126.com

Table of Contents

1. ^{13}C NMR spectral data··S1

2. X-ray Diffraction···S3

3. Thermal Behaviors···S19

4. Computation Details···S21

5. References··S22

1. ^{13}C NMR spectral data

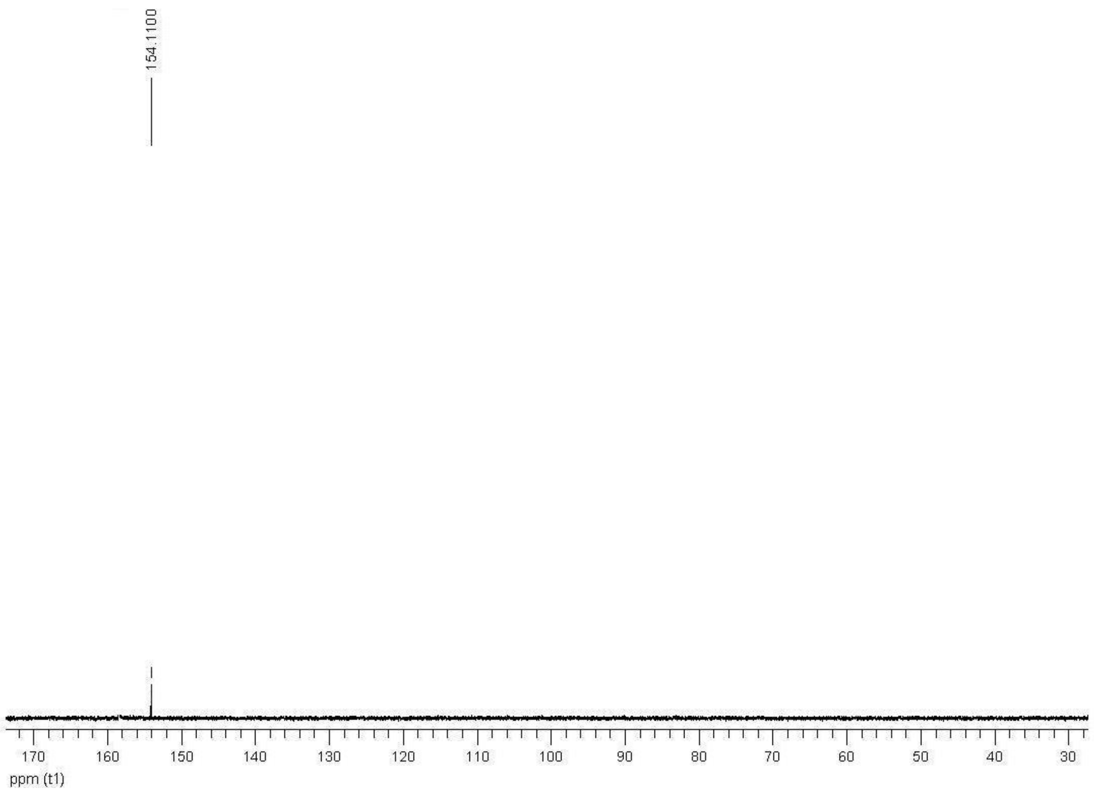

Figure S1. ^{13}C NMR spectrum (125 MHz) of compound **1** in D_2O at 25 ^oC

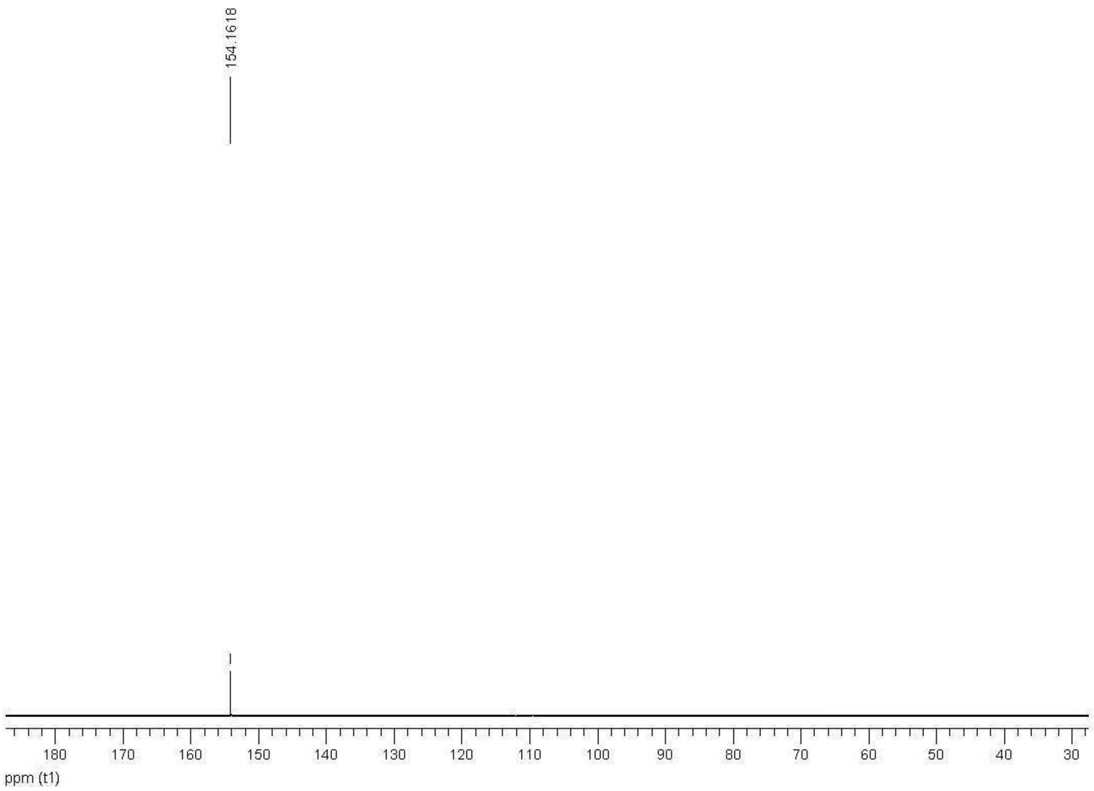

Figure S2. ^{13}C NMR spectrum (125 MHz) of compound **2** in D_2O at 25 ^oC

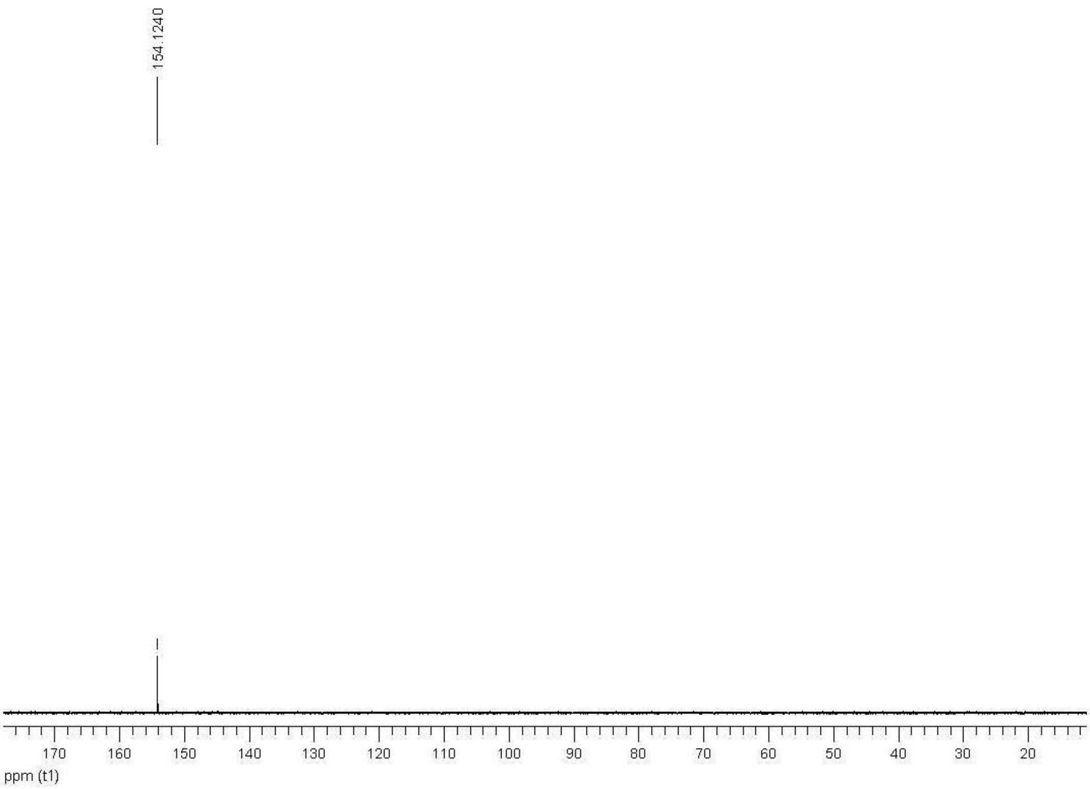

Figure S3. ^{13}C NMR spectrum (125 MHz) of compound **3** in D_2O at 25 ^oC

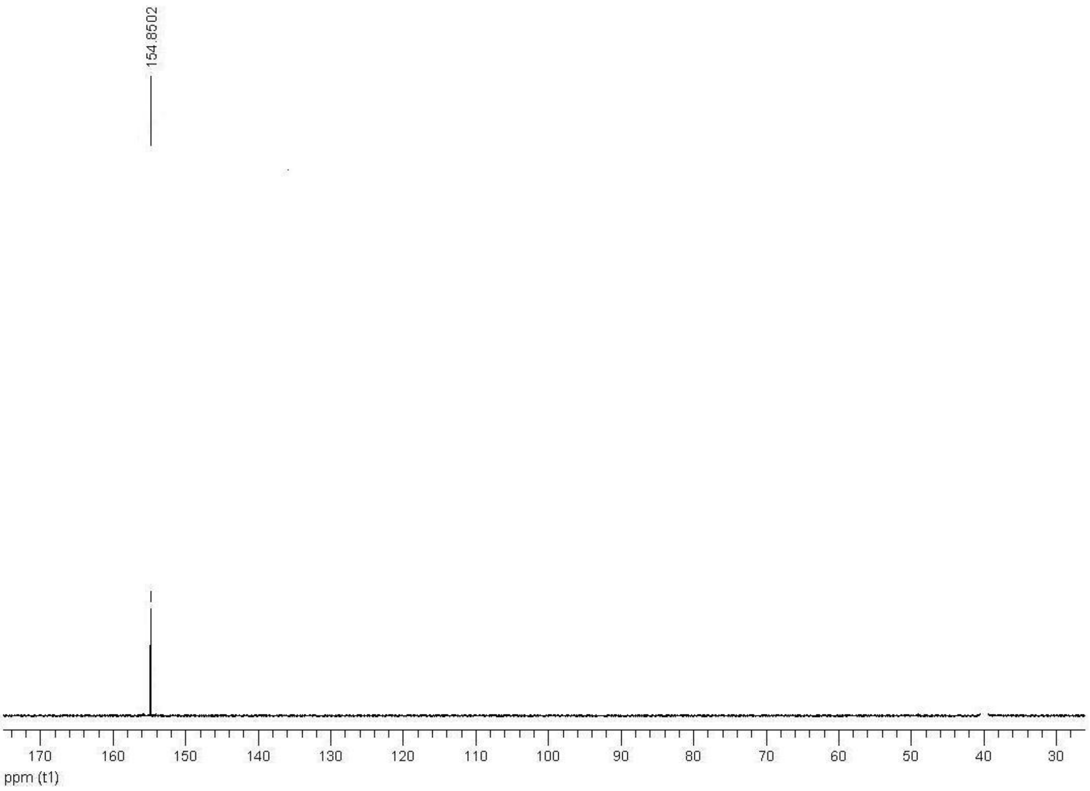

Figure S4. ^{13}C NMR spectrum (125 MHz) of compound **4** in D_2O at 25 ^oC

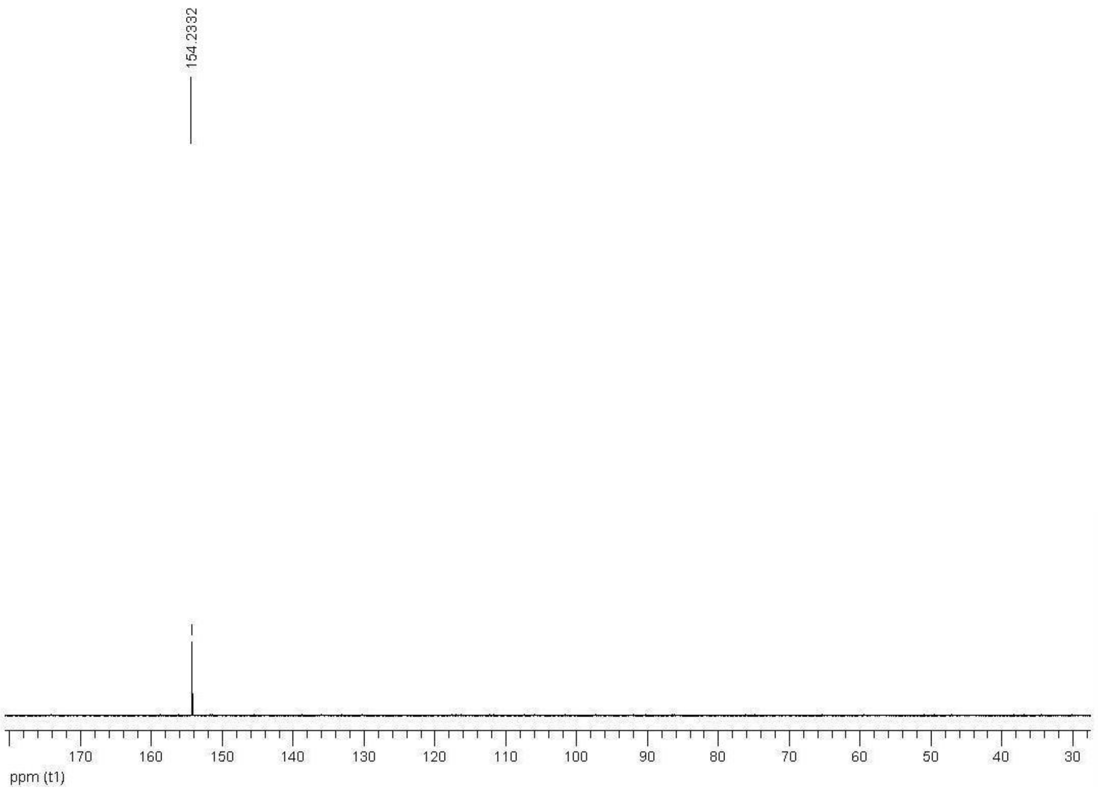

Figure S5. ^{13}C NMR spectrum (125 MHz) of compound **5** in D_2O at 25 °C

2. X-ray Diffraction

For all target compounds **1~5**, a Bruker SMART Apex II X-ray diffractometer with a CCD area detector was employed for data collection at 296 K using Mo-Kα radiation (λ = 0.71073 Å). The structures were solved by direct methods and refined by full-matrix least-squares techniques on F^2 using SHELXL-97 programs.[4] The non-hydrogen atoms were refined anisotropically and the hydrogen atoms were located and freely refined. Crystal data such as bond lengths and bond angles are summarized in Table S1~S5. The crystal structures have been deposited in the Cambridge Crystallographic Data Centre with CCDC 1826491, 1826485, 1826494, 1826488, and 1848490 for compounds **1~5**, respectively. These data can be obtained from The Cambridge Crystallographic Data Centre via www.ccdc.cam.ac.uk/data _ request/cif.

Table S1. Bond lengths [Å] and angles [°] for compound **1**

**Bond lengths**	**Å**	**Bond angles**	**°**
Fe(1)-N(3)	2.0899(13)	N(3)-Fe(1)-N(3)#1	179.999(1)
Fe(1)-N(3)#1	2.0899(13)	N(3)-Fe(1)-O(1)#1	90.71(5)
Fe(1)-O(1)#1	2.1321(13)	N(3)#1-Fe(1)-O(1)#1	89.29(5)
Fe(1)-O(1)	2.1321(13)	N(3)-Fe(1)-O(1)	89.29(5)
Fe(1)-O(2)	2.1634(12)	N(3)#1-Fe(1)-O(1)	90.71(5)
Fe(1)-O(2)#1	2.1634(12)	O(1)#1-Fe(1)-O(1)	180.00(5)
K(1)-O(5)	2.6947(14)	N(3)-Fe(1)-O(2)	78.15(5)
K(1)-O(4)#2	2.8178(14)	N(3)#1-Fe(1)-O(2)	101.85(5)
K(1)-O(6)	2.8211(14)	O(1)#1-Fe(1)-O(2)	90.52(5)
K(1)-O(3)#3	2.8575(13)	O(1)-Fe(1)-O(2)	89.48(5)

K(1)-O(4)#4	2.8934(15)	N(3)-Fe(1)-O(2)#1	101.85(5)
K(1)-O(5)#4	2.9837(15)	N(3)#1-Fe(1)-O(2)#1	78.15(5)
K(1)-O(3)#5	3.0358(14)	O(1)#1-Fe(1)-O(2)#1	89.48(5)
K(1)-O(1)#6	3.1379(15)	O(1)-Fe(1)-O(2)#1	90.52(5)
K(1)-N(8)#4	3.3154(15)	O(2)-Fe(1)-O(2)#1	180.0
K(1)-N(5)#2	3.3719(16)	O(5)-K(1)-O(4)#2	147.11(5)
K(1)-K(1)#7	4.0180(9)	O(5)-K(1)-O(6)	68.97(4)
K(1)-K(1)#4	4.4820(9)	O(4)#2-K(1)-O(6)	79.17(4)
K(1)-H(6A)	2.98(3)	O(5)-K(1)-O(3)#3	140.41(4)
N(1)-O(3)	1.2415(17)	O(4)#2-K(1)-O(3)#3	66.30(4)
N(1)-O(2)	1.2685(17)	O(6)-K(1)-O(3)#3	142.50(4)
N(1)-N(2)	1.3168(19)	O(5)-K(1)-O(4)#4	118.76(4)
N(2)-C(1)	1.365(2)	O(4)#2-K(1)-O(4)#4	90.59(4)
N(3)-C(1)	1.328(2)	O(6)-K(1)-O(4)#4	138.96(4)
N(3)-N(4)	1.3597(19)	O(3)#3-K(1)-O(4)#4	58.58(4)
N(4)-N(5)	1.284(2)	O(5)-K(1)-O(5)#4	75.87(4)
N(5)-N(6)	1.351(2)	O(4)#2-K(1)-O(5)#4	129.62(4)
N(5)-K(1)#8	3.3720(16)	O(6)-K(1)-O(5)#4	119.69(4)
N(6)-C(1)	1.345(2)	O(3)#3-K(1)-O(5)#4	94.38(4)
N(6)-N(7)	1.3907(19)	O(4)#4-K(1)-O(5)#4	43.18(4)
N(7)-N(8)	1.332(2)	O(5)-K(1)-O(3)#5	120.83(4)
N(8)-O(4)	1.2379(19)	O(4)#2-K(1)-O(3)#5	57.31(4)
N(8)-O(5)	1.2442(19)	O(6)-K(1)-O(3)#5	78.34(4)
N(8)-K(1)#4	3.3154(15)	O(3)#3-K(1)-O(3)#5	94.09(4)
O(1)-K(1)#9	3.1380(15)	O(4)#4-K(1)-O(3)#5	63.08(4)
O(1)-H(1A)	0.802(17)	O(5)#4-K(1)-O(3)#5	79.92(4)
O(1)-H(1B)	0.825(16)	O(5)-K(1)-O(1)#6	77.65(4)
O(3)-K(1)#10	2.8575(13)	O(4)#2-K(1)-O(1)#6	111.55(4)
O(3)-K(1)#11	3.0358(14)	O(6)-K(1)-O(1)#6	117.26(4)
O(4)-K(1)#8	2.8179(14)	O(3)#3-K(1)-O(1)#6	66.30(4)
O(4)-K(1)#4	2.8935(15)	O(4)#4-K(1)-O(1)#6	103.51(4)
O(5)-K(1)#4	2.9837(15)	O(5)#4-K(1)-O(1)#6	100.20(4)
O(6)-H(6B)	0.824(16)	O(3)#5-K(1)-O(1)#6	160.38(4)
O(6)-H(6A)	0.811(17)	O(5)-K(1)-N(8)#4	97.84(4)
		O(4)#2-K(1)-N(8)#4	108.88(4)
		O(6)-K(1)-N(8)#4	129.43(4)
		O(3)#3-K(1)-N(8)#4	77.81(4)
		O(4)#4-K(1)-N(8)#4	21.66(4)
		O(5)#4-K(1)-N(8)#4	21.98(3)
		O(3)#5-K(1)-N(8)#4	67.25(4)
		O(1)#6-K(1)-N(8)#4	105.99(4)
		O(5)-K(1)-N(5)#2	106.49(4)
		O(4)#2-K(1)-N(5)#2	54.45(4)
		O(6)-K(1)-N(5)#2	73.10(4)
		O(3)#3-K(1)-N(5)#2	75.07(4)
		O(4)#4-K(1)-N(5)#2	131.06(4)
		O(5)#4-K(1)-N(5)#2	166.23(4)
		O(3)#5-K(1)-N(5)#2	109.21(4)
		O(1)#6-K(1)-N(5)#2	67.66(4)
		N(8)#4-K(1)-N(5)#2	152.33(4)
		O(5)-K(1)-K(1)#7	159.29(4)
		O(4)#2-K(1)-K(1)#7	46.06(3)
		O(6)-K(1)-K(1)#7	114.30(3)
		O(3)#3-K(1)-K(1)#7	48.91(3)
		O(4)#4-K(1)-K(1)#7	44.53(3)

		O(5)#4-K(1)-K(1)#7	85.53(3)
		O(3)#5-K(1)-K(1)#7	45.18(3)
		O(1)#6-K(1)-K(1)#7	115.21(3)
		N(8)#4-K(1)-K(1)#7	63.74(3)
		N(5)#2-K(1)-K(1)#7	93.75(3)
		O(5)-K(1)-K(1)#4	40.21(3)
		O(4)#2-K(1)-K(1)#4	158.34(3)
		O(6)-K(1)-K(1)#4	96.54(3)
		O(3)#3-K(1)-K(1)#4	120.94(3)
		O(4)#4-K(1)-K(1)#4	78.69(3)
		O(5)#4-K(1)-K(1)#4	35.66(2)
		O(3)#5-K(1)-K(1)#4	101.05(3)
		O(1)#6-K(1)-K(1)#4	89.38(3)
		N(8)#4-K(1)-K(1)#4	57.64(3)
		N(5)#2-K(1)-K(1)#4	144.81(3)
		K(1)#7-K(1)-K(1)#4	120.70(2)
		O(5)-K(1)-H(6A)	57.7(4)
		O(4)#2-K(1)-H(6A)	92.4(4)
		O(6)-K(1)-H(6A)	15.8(3)
		O(3)#3-K(1)-H(6A)	157.8(4)
		O(4)#4-K(1)-H(6A)	131.3(5)
		O(5)#4-K(1)-H(6A)	104.5(4)
		O(3)#5-K(1)-H(6A)	78.2(5)
		O(1)#6-K(1)-H(6A)	120.2(5)
		N(8)#4-K(1)-H(6A)	116.9(4)
		N(5)#2-K(1)-H(6A)	87.8(4)
		K(1)#7-K(1)-H(6A)	120.3(5)
		K(1)#4-K(1)-H(6A)	81.1(4)
		O(3)-N(1)-O(2)	118.56(13)
		O(3)-N(1)-N(2)	116.59(13)
		O(2)-N(1)-N(2)	124.84(13)
		N(1)-N(2)-C(1)	116.92(13)
		C(1)-N(3)-N(4)	106.60(13)
		C(1)-N(3)-Fe(1)	125.46(11)
		N(4)-N(3)-Fe(1)	127.94(10)
		N(5)-N(4)-N(3)	110.96(13)
		N(4)-N(5)-N(6)	106.20(13)
		N(4)-N(5)-K(1)#8	96.89(10)
		N(6)-N(5)-K(1)#8	107.58(10)
		C(1)-N(6)-N(5)	109.33(13)
		C(1)-N(6)-N(7)	127.38(14)
		N(5)-N(6)-N(7)	122.85(13)
		N(8)-N(7)-N(6)	109.23(13)
		O(4)-N(8)-O(5)	121.36(15)
		O(4)-N(8)-N(7)	123.13(15)
		O(5)-N(8)-N(7)	115.51(14)
		O(4)-N(8)-K(1)#4	59.61(9)
		O(5)-N(8)-K(1)#4	63.84(9)
		N(7)-N(8)-K(1)#4	164.74(11)
		Fe(1)-O(1)-K(1)#9	123.09(6)
		Fe(1)-O(1)-H(1A)	120(2)
		K(1)#9-O(1)-H(1A)	90(2)
		Fe(1)-O(1)-H(1B)	114.5(17)
		K(1)#9-O(1)-H(1B)	98.5(17)
		H(1A)-O(1)-H(1B)	107(3)

		N(1)-O(2)-Fe(1)	129.95(10)
		N(1)-O(3)-K(1)#10	130.42(10)
		N(1)-O(3)-K(1)#11	109.40(10)
		K(1)#10-O(3)-K(1)#11	85.91(4)
		N(8)-O(4)-K(1)#8	147.40(11)
		N(8)-O(4)-K(1)#4	98.73(10)
		K(1)#8-O(4)-K(1)#4	89.41(4)
		N(8)-O(5)-K(1)	161.59(12)
		N(8)-O(5)-K(1)#4	94.18(10)
		K(1)-O(5)-K(1)#4	104.13(4)
		K(1)-O(6)-H(6B)	114.2(17)
		K(1)-O(6)-H(6A)	93.2(19)
		H(6B)-O(6)-H(6A)	105(2)
		N(3)-C(1)-N(6)	106.91(14)
		N(3)-C(1)-N(2)	133.49(15)
		N(6)-C(1)-N(2)	119.53(14)

Table S2. Bond lengths [Å] and angles [°] for compound **2**

**Bond lengths**	**Å**	**Bond angles**	**°**
Cu(1)-N(3)	1.945(3)	N(3)-Cu(1)-N(3)#1	180.0
Cu(1)-N(3)#1	1.945(3)	N(3)-Cu(1)-O(5)	90.36(14)
Cu(1)-O(5)	2.072(3)	N(3)#1-Cu(1)-O(5)	89.64(14)
Cu(1)-O(5)#1	2.072(4)	N(3)-Cu(1)-O(5)#1	89.64(14)
Cu(1)-O(1)#1	2.273(3)	N(3)#1-Cu(1)-O(5)#1	90.36(14)
Cu(1)-O(1)	2.273(3)	O(5)-Cu(1)-O(5)#1	179.999(1)
N(7)-N(8)	1.338(5)	N(3)-Cu(1)-O(1)#1	101.82(13)
N(7)-N(6)	1.390(5)	N(3)#1-Cu(1)-O(1)#1	78.18(13)
O(4)-N(8)	1.243(5)	O(5)-Cu(1)-O(1)#1	90.24(14)
O(4)-K(1)#2	2.691(3)	O(5)#1-Cu(1)-O(1)#1	89.76(14)
O(4)-K(1)#3	2.996(4)	N(3)-Cu(1)-O(1)	78.18(13)
N(8)-O(3)	1.234(5)	N(3)#1-Cu(1)-O(1)	101.82(13)
N(8)-K(1)#3	3.323(4)	O(5)-Cu(1)-O(1)	89.76(14)
N(2)-N(1)	1.325(5)	O(5)#1-Cu(1)-O(1)	90.24(14)
N(2)-C(1)	1.352(5)	O(1)#1-Cu(1)-O(1)	180.0
N(1)-O(2)	1.244(4)	N(8)-N(7)-N(6)	108.8(3)
N(1)-O(1)	1.254(4)	N(8)-O(4)-K(1)#2	162.8(3)
O(5)-K(1)#4	3.180(4)	N(8)-O(4)-K(1)#3	94.0(3)
O(5)-H(5A)	0.82(2)	K(1)#2-O(4)-K(1)#3	103.15(10)
O(5)-H(5B)	0.81(2)	O(3)-N(8)-O(4)	121.3(4)
N(4)-N(5)	1.286(5)	O(3)-N(8)-N(7)	123.2(4)
N(4)-N(3)	1.368(5)	O(4)-N(8)-N(7)	115.5(4)
O(2)-K(1)#5	2.815(3)	O(3)-N(8)-K(1)#3	59.2(2)
O(2)-K(1)#6	3.049(4)	O(4)-N(8)-K(1)#3	64.1(2)
N(3)-C(1)	1.340(5)	N(7)-N(8)-K(1)#3	165.3(3)
C(1)-N(6)	1.346(5)	N(1)-N(2)-C(1)	116.7(3)
N(5)-N(6)	1.348(5)	O(2)-N(1)-O(1)	119.5(4)
N(5)-K(1)	3.435(4)	O(2)-N(1)-N(2)	116.0(3)
O(3)-K(1)	2.835(4)	O(1)-N(1)-N(2)	124.5(3)
O(3)-K(1)#3	2.891(4)	Cu(1)-O(5)-K(1)#4	124.65(15)
O(1)-K(1)#6	3.336(3)	Cu(1)-O(5)-H(5A)	116(5)
O(6)-K(1)	2.811(4)	K(1)#4-O(5)-H(5A)	97(5)
O(6)-H(6A)	0.82(2)	Cu(1)-O(5)-H(5B)	117(5)
O(6)-H(6B)	0.82(2)	K(1)#4-O(5)-H(5B)	89(5)
K(1)-O(4)#7	2.691(3)	H(5A)-O(5)-H(5B)	109(6)
K(1)-O(2)#8	2.815(3)	N(5)-N(4)-N(3)	110.4(3)

K(1)-O(3)#3	2.891(4)	N(1)-O(2)-K(1)#5	130.3(3)
K(1)-O(4)#3	2.996(4)	N(1)-O(2)-K(1)#6	105.4(3)
K(1)-O(2)#9	3.049(4)	K(1)#5-O(2)-K(1)#6	86.32(10)
K(1)-O(5)#10	3.180(4)	C(1)-N(3)-N(4)	107.0(3)
K(1)-N(8)#3	3.323(4)	C(1)-N(3)-Cu(1)	128.9(3)
K(1)-O(1)#9	3.336(3)	N(4)-N(3)-Cu(1)	124.0(3)
K(1)-K(1)#3	4.015(2)	N(3)-C(1)-N(6)	106.0(3)
K(1)-H(6B)	2.97(6)	N(3)-C(1)-N(2)	134.0(4)
		N(6)-C(1)-N(2)	120.0(3)
		N(4)-N(5)-N(6)	106.5(3)
		N(4)-N(5)-K(1)	96.7(3)
		N(6)-N(5)-K(1)	106.5(3)
		N(8)-O(3)-K(1)	148.5(3)
		N(8)-O(3)-K(1)#3	99.3(2)
		K(1)-O(3)-K(1)#3	89.05(10)
		N(1)-O(1)-Cu(1)	126.9(3)
		N(1)-O(1)-K(1)#6	91.1(2)
		Cu(1)-O(1)-K(1)#6	140.64(12)
		C(1)-N(6)-N(5)	110.1(3)
		C(1)-N(6)-N(7)	126.2(3)
		N(5)-N(6)-N(7)	123.2(3)
		K(1)-O(6)-H(6A)	113(4)
		K(1)-O(6)-H(6B)	93(4)
		H(6A)-O(6)-H(6B)	108(6)
		O(4)#7-K(1)-O(6)	69.53(11)
		O(4)#7-K(1)-O(2)#8	139.40(12)
		O(6)-K(1)-O(2)#8	143.24(11)
		O(4)#7-K(1)-O(3)	146.51(11)
		O(6)-K(1)-O(3)	78.69(11)
		O(2)#8-K(1)-O(3)	66.90(11)
		O(4)#7-K(1)-O(3)#3	119.52(11)
		O(6)-K(1)-O(3)#3	137.28(11)
		O(2)#8-K(1)-O(3)#3	58.57(10)
		O(3)-K(1)-O(3)#3	90.95(10)
		O(4)#7-K(1)-O(4)#3	76.85(10)
		O(6)-K(1)-O(4)#3	119.09(11)
		O(2)#8-K(1)-O(4)#3	93.72(10)
		O(3)-K(1)-O(4)#3	130.07(10)
		O(3)#3-K(1)-O(4)#3	42.98(9)
		O(4)#7-K(1)-O(2)#9	122.79(11)
		O(6)-K(1)-O(2)#9	76.97(10)
		O(2)#8-K(1)-O(2)#9	93.68(10)
		O(3)-K(1)-O(2)#9	56.52(9)
		O(3)#3-K(1)-O(2)#9	63.17(10)

		O(4)#3-K(1)-O(2)#9	81.07(10)
		O(4)#7-K(1)-O(5)#10	77.22(11)
		O(6)-K(1)-O(5)#10	119.13(10)
		O(2)#8-K(1)-O(5)#10	65.49(10)
		O(3)-K(1)-O(5)#10	110.97(10)
		O(3)#3-K(1)-O(5)#10	103.35(10)
		O(4)#3-K(1)-O(5)#10	99.90(10)
		O(2)#9-K(1)-O(5)#10	159.16(9)
		O(4)#7-K(1)-N(8)#3	98.74(10)
		O(6)-K(1)-N(8)#3	128.31(10)
		O(2)#8-K(1)-N(8)#3	77.39(9)
		O(3)-K(1)-N(8)#3	109.33(10)
		O(3)#3-K(1)-N(8)#3	21.49(9)
		O(4)#3-K(1)-N(8)#3	21.91(8)
		O(2)#9-K(1)-N(8)#3	68.09(9)
		O(5)#10-K(1)-N(8)#3	105.60(9)
		O(4)#7-K(1)-O(1)#9	85.08(10)
		O(6)-K(1)-O(1)#9	69.00(10)
		O(2)#8-K(1)-O(1)#9	123.74(10)
		O(3)-K(1)-O(1)#9	93.40(10)
		O(3)#3-K(1)-O(1)#9	70.44(10)
		O(4)#3-K(1)-O(1)#9	58.65(10)
		O(2)#9-K(1)-O(1)#9	39.17(8)
		O(5)#10-K(1)-O(1)#9	155.17(9)
		N(8)#3-K(1)-O(1)#9	59.74(9)
		O(4)#7-K(1)-N(5)	104.77(10)
		O(6)-K(1)-N(5)	72.43(10)
		O(2)#8-K(1)-N(5)	76.94(9)
		O(3)-K(1)-N(5)	53.89(9)
		O(3)#3-K(1)-N(5)	132.41(10)
		O(4)#3-K(1)-N(5)	167.52(10)
		O(2)#9-K(1)-N(5)	107.48(9)
		O(5)#10-K(1)-N(5)	68.82(9)
		N(8)#3-K(1)-N(5)	153.57(10)
		O(1)#9-K(1)-N(5)	133.54(10)
		O(4)#7-K(1)-K(1)#3	160.66(9)
		O(6)-K(1)-K(1)#3	112.99(8)
		O(2)#8-K(1)-K(1)#3	49.27(8)
		O(3)-K(1)-K(1)#3	46.06(7)
		O(3)#3-K(1)-K(1)#3	44.90(7)
		O(4)#3-K(1)-K(1)#3	85.85(7)
		O(2)#9-K(1)-K(1)#3	44.40(7)
		O(5)#10-K(1)-K(1)#3	114.76(7)

		N(8)#3-K(1)-K(1)#3	64.13(7)
		O(1)#9-K(1)-K(1)#3	78.51(6)
		N(5)-K(1)-K(1)#3	93.99(7)
		O(4)#7-K(1)-H(6B)	59.1(10)
		O(6)-K(1)-H(6B)	15.9(5)
		O(2)#8-K(1)-H(6B)	158.3(7)
		O(3)-K(1)-H(6B)	91.7(8)
		O(3)#3-K(1)-H(6B)	128.9(10)
		O(4)#3-K(1)-H(6B)	103.6(6)
		O(2)#9-K(1)-H(6B)	76.5(12)
		O(5)#10-K(1)-H(6B)	122.8(12)
		N(8)#3-K(1)-H(6B)	115.3(8)
		O(1)#9-K(1)-H(6B)	58.5(10)
		N(5)-K(1)-H(6B)	87.5(6)
		K(1)#3-K(1)-H(6B)	118.3(12)

Table S3. Bond lengths [Å] and angles [°] for compound **3**

**Bond lengths**	**Å**	**Bond angles**	**°**
K(1)-O(1)#1	2.687(2)	O(1)#1-K(1)-O(5)	139.09(7)
K(1)-O(5)	2.817(2)	O(1)#1-K(1)-O(6)	69.50(7)
K(1)-O(6)	2.818(2)	O(5)-K(1)-O(6)	143.38(7)
K(1)-O(2)#2	2.837(3)	O(1)#1-K(1)-O(2)#2	146.63(8)
K(1)-O(2)#3	2.891(3)	O(5)-K(1)-O(2)#2	66.89(7)
K(1)-O(1)#3	3.003(3)	O(6)-K(1)-O(2)#2	78.91(7)
K(1)-O(5)#4	3.046(2)	O(1)#1-K(1)-O(2)#3	119.54(7)
K(1)-O(3)#5	3.192(3)	O(5)-K(1)-O(2)#3	58.52(6)
K(1)-O(4)#4	3.321(2)	O(6)-K(1)-O(2)#3	137.58(7)
K(1)-N(1)#3	3.324(3)	O(2)#2-K(1)-O(2)#3	91.00(7)
K(1)-N(4)#2	3.447(3)	O(1)#1-K(1)-O(1)#3	76.69(7)
K(1)-K(1)#4	4.0152(16)	O(5)-K(1)-O(1)#3	93.73(6)
K(1)-H(6B)	2.96(4)	O(6)-K(1)-O(1)#3	119.18(7)
Ni(1)-N(6)#6	1.953(2)	O(2)#2-K(1)-O(1)#3	130.38(6)
Ni(1)-N(6)	1.953(2)	O(2)#3-K(1)-O(1)#3	43.16(6)
Ni(1)-O(3)	2.068(2)	O(1)#1-K(1)-O(5)#4	123.14(7)
Ni(1)-O(3)#6	2.068(2)	O(5)-K(1)-O(5)#4	93.65(7)
Ni(1)-O(4)	2.288(2)	O(6)-K(1)-O(5)#4	77.25(6)
Ni(1)-O(4)#6	2.288(2)	O(2)#2-K(1)-O(5)#4	56.51(6)
N(1)-O(2)	1.242(3)	O(2)#3-K(1)-O(5)#4	63.26(7)
N(1)-O(1)	1.245(3)	O(1)#3-K(1)-O(5)#4	81.42(6)
N(1)-N(2)	1.339(3)	O(1)#1-K(1)-O(3)#5	77.09(7)
N(1)-K(1)#7	3.324(3)	O(5)-K(1)-O(3)#5	65.30(6)
N(2)-N(3)	1.396(3)	O(6)-K(1)-O(3)#5	119.00(6)
N(3)-C(1)	1.345(3)	O(2)#2-K(1)-O(3)#5	110.79(7)

N(3)-N(4)	1.346(3)	O(2)#3-K(1)-O(3)#5	103.17(6)
N(4)-N(5)	1.295(3)	O(1)#3-K(1)-O(3)#5	99.65(6)
N(4)-K(1)#8	3.447(3)	O(5)#4-K(1)-O(3)#5	158.95(6)
N(5)-N(6)	1.360(3)	O(1)#1-K(1)-O(4)#4	85.37(6)
N(6)-C(1)	1.338(3)	O(5)-K(1)-O(4)#4	123.62(6)
N(7)-N(8)	1.331(3)	O(6)-K(1)-O(4)#4	69.39(6)
N(7)-C(1)	1.363(3)	O(2)#2-K(1)-O(4)#4	93.58(6)
N(8)-O(5)	1.237(3)	O(2)#3-K(1)-O(4)#4	70.30(6)
N(8)-O(4)	1.257(3)	O(1)#3-K(1)-O(4)#4	58.65(6)
O(1)-K(1)#9	2.687(2)	O(5)#4-K(1)-O(4)#4	39.29(5)
O(1)-K(1)#7	3.003(3)	O(3)#5-K(1)-O(4)#4	155.10(6)
O(2)-K(1)#8	2.837(3)	O(1)#1-K(1)-N(1)#3	98.62(7)
O(2)-K(1)#7	2.891(3)	O(5)-K(1)-N(1)#3	77.44(6)
O(3)-K(1)#10	3.192(3)	O(6)-K(1)-N(1)#3	128.49(7)
O(3)-H(3A)	0.821(10)	O(2)#2-K(1)-N(1)#3	109.56(7)
O(3)-H(3B)	0.822(10)	O(2)#3-K(1)-N(1)#3	21.65(6)
O(4)-K(1)#4	3.321(2)	O(1)#3-K(1)-N(1)#3	21.94(6)
O(5)-K(1)#4	3.046(2)	O(5)#4-K(1)-N(1)#3	68.32(6)
O(6)-H(6B)	0.820(10)	O(3)#5-K(1)-N(1)#3	105.41(6)
O(6)-H(6A)	0.817(10)	O(4)#4-K(1)-N(1)#3	59.59(6)
		O(1)#1-K(1)-N(4)#2	104.81(7)
		O(5)-K(1)-N(4)#2	76.88(6)
		O(6)-K(1)-N(4)#2	72.38(6)
		O(2)#2-K(1)-N(4)#2	53.67(6)
		O(2)#3-K(1)-N(4)#2	132.27(6)
		O(1)#3-K(1)-N(4)#2	167.40(7)
		O(5)#4-K(1)-N(4)#2	107.26(6)
		O(3)#5-K(1)-N(4)#2	68.92(6)
		O(4)#4-K(1)-N(4)#2	133.67(6)
		N(1)#3-K(1)-N(4)#2	153.58(6)
		O(1)#1-K(1)-K(1)#4	160.91(6)
		O(5)-K(1)-K(1)#4	49.21(5)
		O(6)-K(1)-K(1)#4	113.31(6)
		O(2)#2-K(1)-K(1)#4	46.05(5)
		O(2)#3-K(1)-K(1)#4	44.94(5)
		O(1)#3-K(1)-K(1)#4	86.13(5)
		O(5)#4-K(1)-K(1)#4	44.45(4)
		O(3)#5-K(1)-K(1)#4	114.51(5)
		O(4)#4-K(1)-K(1)#4	78.54(4)
		N(1)#3-K(1)-K(1)#4	64.36(5)
		N(4)#2-K(1)-K(1)#4	93.77(5)
		O(1)#1-K(1)-H(6B)	59.6(6)
		O(5)-K(1)-H(6B)	158.3(5)

		O(6)-K(1)-H(6B)	16.1(3)
		O(2)#2-K(1)-H(6B)	91.7(5)
		O(2)#3-K(1)-H(6B)	128.6(6)
		O(1)#3-K(1)-H(6B)	103.4(3)
		O(5)#4-K(1)-H(6B)	76.1(7)
		O(3)#5-K(1)-H(6B)	123.4(7)
		O(4)#4-K(1)-H(6B)	58.3(6)
		N(1)#3-K(1)-H(6B)	114.9(5)
		N(4)#2-K(1)-H(6B)	87.8(4)
		K(1)#4-K(1)-H(6B)	118.0(7)
		N(6)#6-Ni(1)-N(6)	180.0
		N(6)#6-Ni(1)-O(3)	89.63(9)
		N(6)-Ni(1)-O(3)	90.37(9)
		N(6)#6-Ni(1)-O(3)#6	90.37(9)
		N(6)-Ni(1)-O(3)#6	89.63(9)
		O(3)-Ni(1)-O(3)#6	180.0
		N(6)#6-Ni(1)-O(4)	101.86(8)
		N(6)-Ni(1)-O(4)	78.14(8)
		O(3)-Ni(1)-O(4)	89.94(9)
		O(3)#6-Ni(1)-O(4)	90.06(9)
		N(6)#6-Ni(1)-O(4)#6	78.14(8)
		N(6)-Ni(1)-O(4)#6	101.86(8)
		O(3)-Ni(1)-O(4)#6	90.06(9)
		O(3)#6-Ni(1)-O(4)#6	89.94(9)
		O(4)-Ni(1)-O(4)#6	180.0
		O(2)-N(1)-O(1)	121.5(2)
		O(2)-N(1)-N(2)	123.0(2)
		O(1)-N(1)-N(2)	115.5(2)
		O(2)-N(1)-K(1)#7	59.16(14)
		O(1)-N(1)-K(1)#7	64.34(15)
		N(2)-N(1)-K(1)#7	165.39(19)
		N(1)-N(2)-N(3)	108.7(2)
		C(1)-N(3)-N(4)	110.1(2)
		C(1)-N(3)-N(2)	126.4(2)
		N(4)-N(3)-N(2)	123.0(2)
		N(5)-N(4)-N(3)	106.4(2)
		N(5)-N(4)-K(1)#8	96.64(17)
		N(3)-N(4)-K(1)#8	106.51(16)
		N(4)-N(5)-N(6)	110.1(2)
		C(1)-N(6)-N(5)	107.4(2)
		C(1)-N(6)-Ni(1)	128.72(18)
		N(5)-N(6)-Ni(1)	123.84(17)
		N(8)-N(7)-C(1)	116.7(2)

		O(5)-N(8)-O(4)	119.7(2)
		O(5)-N(8)-N(7)	116.3(2)
		O(4)-N(8)-N(7)	124.0(2)
		N(1)-O(1)-K(1)#9	162.9(2)
		N(1)-O(1)-K(1)#7	93.73(17)
		K(1)#9-O(1)-K(1)#7	103.31(7)
		N(1)-O(2)-K(1)#8	148.78(19)
		N(1)-O(2)-K(1)#7	99.19(17)
		K(1)#8-O(2)-K(1)#7	89.00(7)
		Ni(1)-O(3)-K(1)#10	124.62(9)
		Ni(1)-O(3)-H(3A)	119(3)
		K(1)#10-O(3)-H(3A)	86(3)
		Ni(1)-O(3)-H(3B)	116(3)
		K(1)#10-O(3)-H(3B)	94(3)
		H(3A)-O(3)-H(3B)	111(4)
		N(8)-O(4)-Ni(1)	127.10(17)
		N(8)-O(4)-K(1)#4	91.05(15)
		Ni(1)-O(4)-K(1)#4	140.39(8)
		N(8)-O(5)-K(1)	129.86(16)
		N(8)-O(5)-K(1)#4	105.02(16)
		K(1)-O(5)-K(1)#4	86.35(7)
		K(1)-O(6)-H(6B)	92(3)
		K(1)-O(6)-H(6A)	115(3)
		H(6B)-O(6)-H(6A)	103(4)
		N(6)-C(1)-N(3)	106.0(2)
		N(6)-C(1)-N(7)	134.3(2)
		N(3)-C(1)-N(7)	119.6(2)

Table S4. Bond lengths [Å] and angles [°] for compound **4**

**Bond lengths**	**Å**	**Bond angles**	**°**
K(1)-O(2)#1	2.6917(17)	O(2)#1-K(1)-O(1)	146.33(6)
K(1)-O(1)	2.8172(18)	O(2)#1-K(1)-O(6)	69.46(5)
K(1)-O(6)	2.8172(18)	O(1)-K(1)-O(6)	77.58(5)
K(1)-O(3)#2	2.8494(17)	O(2)#1-K(1)-O(3)#2	142.05(5)
K(1)-O(1)#3	2.8932(19)	O(1)-K(1)-O(3)#2	66.42(5)
K(1)-O(2)#3	2.974(2)	O(6)-K(1)-O(3)#2	141.33(5)
K(1)-O(3)#4	3.0427(18)	O(2)#1-K(1)-O(1)#3	118.63(5)
K(1)-O(5)#5	3.1152(19)	O(1)-K(1)-O(1)#3	90.47(5)
K(1)-N(1)#3	3.307(2)	O(6)-K(1)-O(1)#3	138.05(5)
K(1)-N(4)	3.343(2)	O(3)#2-K(1)-O(1)#3	58.51(5)
K(1)-K(1)#3	4.0215(11)	O(2)#1-K(1)-O(2)#3	75.72(6)
K(1)-K(1)#6	4.4764(11)	O(1)-K(1)-O(2)#3	128.74(5)
K(1)-H(6B)	2.95(3)	O(6)-K(1)-O(2)#3	119.12(5)
Co(1)-N(6)#7	2.0459(16)	O(3)#2-K(1)-O(2)#3	95.08(5)

Co(1)-N(6)	2.0459(17)	O(1)#3-K(1)-O(2)#3	43.22(5)
Co(1)-O(5)#7	2.1102(16)	O(2)#1-K(1)-O(3)#4	119.19(5)
Co(1)-O(5)	2.1102(16)	O(1)-K(1)-O(3)#4	57.08(5)
Co(1)-O(4)#7	2.1232(15)	O(6)-K(1)-O(3)#4	77.11(5)
Co(1)-O(4)	2.1232(15)	O(3)#2-K(1)-O(3)#4	93.98(5)
N(1)-O(1)	1.236(2)	O(1)#3-K(1)-O(3)#4	62.99(5)
N(1)-O(2)	1.243(2)	O(2)#3-K(1)-O(3)#4	78.67(5)
N(1)-N(2)	1.332(3)	O(2)#1-K(1)-O(5)#5	78.68(5)
N(1)-K(1)#3	3.307(2)	O(1)-K(1)-O(5)#5	112.64(5)
N(2)-N(3)	1.392(2)	O(6)-K(1)-O(5)#5	118.41(5)
N(3)-C(1)	1.343(3)	O(3)#2-K(1)-O(5)#5	66.86(5)
N(3)-N(4)	1.350(2)	O(1)#3-K(1)-O(5)#5	103.37(5)
N(4)-N(5)	1.282(3)	O(2)#3-K(1)-O(5)#5	100.86(5)
N(5)-N(6)	1.359(2)	O(3)#4-K(1)-O(5)#5	160.80(5)
N(6)-C(1)	1.329(3)	O(2)#1-K(1)-N(1)#3	97.70(5)
N(7)-N(8)	1.318(2)	O(1)-K(1)-N(1)#3	108.25(5)
N(7)-C(1)	1.364(3)	O(6)-K(1)-N(1)#3	128.48(5)
N(8)-O(3)	1.241(2)	O(3)#2-K(1)-N(1)#3	78.17(5)
N(8)-O(4)	1.267(2)	O(1)#3-K(1)-N(1)#3	21.71(4)
O(1)-K(1)#3	2.8932(19)	O(2)#3-K(1)-N(1)#3	22.01(4)
O(2)-K(1)#8	2.6917(17)	O(3)#4-K(1)-N(1)#3	66.33(5)
O(2)-K(1)#3	2.974(2)	O(5)#5-K(1)-N(1)#3	106.44(4)
O(3)-K(1)#9	2.8494(17)	O(2)#1-K(1)-N(4)	106.61(5)
O(3)-K(1)#10	3.0427(18)	O(1)-K(1)-N(4)	55.23(5)
O(5)-K(1)#11	3.1152(19)	O(6)-K(1)-N(4)	72.61(5)
O(5)-H(5A)	0.815(18)	O(3)#2-K(1)-N(4)	75.45(5)
O(5)-H(5B)	0.814(19)	O(1)#3-K(1)-N(4)	131.52(5)
O(6)-H(6A)	0.813(17)	O(2)#3-K(1)-N(4)	167.48(5)
O(6)-H(6B)	0.820(18)	O(3)#4-K(1)-N(4)	109.73(5)
		O(5)#5-K(1)-N(4)	68.07(4)
		N(1)#3-K(1)-N(4)	152.99(5)
		O(2)#1-K(1)-K(1)#3	158.09(5)
		O(1)-K(1)-K(1)#3	46.00(4)
		O(6)-K(1)-K(1)#3	112.61(4)
		O(3)#2-K(1)-K(1)#3	49.00(4)
		O(1)#3-K(1)-K(1)#3	44.47(3)
		O(2)#3-K(1)-K(1)#3	85.07(4)
		O(3)#4-K(1)-K(1)#3	44.98(3)
		O(5)#5-K(1)-K(1)#3	115.85(4)
		N(1)#3-K(1)-K(1)#3	63.32(3)
		N(4)-K(1)-K(1)#3	94.44(4)
		O(2)#1-K(1)-K(1)#6	40.07(4)
		O(1)-K(1)-K(1)#6	156.38(4)

		O(6)-K(1)-K(1)#6	96.45(4)
		O(3)#2-K(1)-K(1)#6	122.21(4)
		O(1)#3-K(1)-K(1)#6	78.70(4)
		O(2)#3-K(1)-K(1)#6	35.64(3)
		O(3)#4-K(1)-K(1)#6	99.37(3)
		O(5)#5-K(1)-K(1)#6	90.41(3)
		N(1)#3-K(1)-K(1)#6	57.64(3)
		N(4)-K(1)-K(1)#6	145.12(4)
		K(1)#3-K(1)-K(1)#6	120.05(3)
		O(2)#1-K(1)-H(6B)	58.5(5)
		O(1)-K(1)-H(6B)	90.5(5)
		O(6)-K(1)-H(6B)	16.2(4)
		O(3)#2-K(1)-H(6B)	156.5(4)
		O(1)#3-K(1)-H(6B)	129.7(6)
		O(2)#3-K(1)-H(6B)	103.4(4)
		O(3)#4-K(1)-H(6B)	75.9(6)
		O(5)#5-K(1)-H(6B)	122.3(6)
		N(1)#3-K(1)-H(6B)	115.4(5)
		N(4)-K(1)-H(6B)	87.9(4)
		K(1)#3-K(1)-H(6B)	117.8(6)
		K(1)#6-K(1)-H(6B)	80.8(4)
		N(6)#7-Co(1)-N(6)	180.0
		N(6)#7-Co(1)-O(5)#7	90.13(6)
		N(6)-Co(1)-O(5)#7	89.86(6)
		N(6)#7-Co(1)-O(5)	89.86(6)
		N(6)-Co(1)-O(5)	90.14(6)
		O(5)#7-Co(1)-O(5)	180.0
		N(6)#7-Co(1)-O(4)#7	79.78(6)
		N(6)-Co(1)-O(4)#7	100.22(6)
		O(5)#7-Co(1)-O(4)#7	89.99(7)
		O(5)-Co(1)-O(4)#7	90.01(7)
		N(6)#7-Co(1)-O(4)	100.22(6)
		N(6)-Co(1)-O(4)	79.78(6)
		O(5)#7-Co(1)-O(4)	90.01(7)
		O(5)-Co(1)-O(4)	89.99(7)
		O(4)#7-Co(1)-O(4)	180.0
		O(1)-N(1)-O(2)	121.40(19)
		O(1)-N(1)-N(2)	123.37(18)
		O(2)-N(1)-N(2)	115.23(18)
		O(1)-N(1)-K(1)#3	59.96(11)
		O(2)-N(1)-K(1)#3	63.74(12)
		N(2)-N(1)-K(1)#3	164.61(14)
		N(1)-N(2)-N(3)	109.07(16)

		C(1)-N(3)-N(4)	109.41(16)
		C(1)-N(3)-N(2)	127.43(17)
		N(4)-N(3)-N(2)	122.65(17)
		N(5)-N(4)-N(3)	106.34(16)
		N(5)-N(4)-K(1)	97.59(13)
		N(3)-N(4)-K(1)	107.66(13)
		N(4)-N(5)-N(6)	110.74(16)
		C(1)-N(6)-N(5)	106.81(17)
		C(1)-N(6)-Co(1)	124.90(14)
		N(5)-N(6)-Co(1)	128.28(13)
		N(8)-N(7)-C(1)	116.84(16)
		O(3)-N(8)-O(4)	118.60(17)
		O(3)-N(8)-N(7)	116.57(16)
		O(4)-N(8)-N(7)	124.82(16)
		N(1)-O(1)-K(1)	144.89(14)
		N(1)-O(1)-K(1)#3	98.32(13)
		K(1)-O(1)-K(1)#3	89.53(5)
		N(1)-O(2)-K(1)#8	161.32(15)
		N(1)-O(2)-K(1)#3	94.25(13)
		K(1)#8-O(2)-K(1)#3	104.28(6)
		N(8)-O(3)-K(1)#9	131.18(13)
		N(8)-O(3)-K(1)#10	110.71(12)
		K(1)#9-O(3)-K(1)#10	86.02(5)
		N(8)-O(4)-Co(1)	129.68(12)
		Co(1)-O(5)-K(1)#11	123.31(7)
		Co(1)-O(5)-H(5A)	114(2)
		K(1)#11-O(5)-H(5A)	96(2)
		Co(1)-O(5)-H(5B)	122(3)
		K(1)#11-O(5)-H(5B)	90(3)
		H(5A)-O(5)-H(5B)	108(3)
		K(1)-O(6)-H(6A)	117.7(19)
		K(1)-O(6)-H(6B)	91(2)
		H(6A)-O(6)-H(6B)	107(3)
		N(6)-C(1)-N(3)	106.71(18)
		N(6)-C(1)-N(7)	133.37(19)
		N(3)-C(1)-N(7)	119.88(18)

Table S5. Bond lengths [Å] and angles [°] for compound **5**

**Bond lengths**	**Å**	**Bond angles**	**°**
Zn(1)-N(3)	2.0199(17)	N(3)-Zn(1)-N(3)#1	180.0
Zn(1)-N(3)#1	2.0199(17)	N(3)-Zn(1)-O(3)	80.04(6)
Zn(1)-O(3)	2.1479(16)	N(3)#1-Zn(1)-O(3)	99.96(6)
Zn(1)-O(3)#1	2.1479(16)	N(3)-Zn(1)-O(3)#1	99.96(6)

Zn(1)-O(4)#1	2.1666(17)	N(3)#1-Zn(1)-O(3)#1	80.04(6)
Zn(1)-O(4)	2.1666(17)	O(3)-Zn(1)-O(3)#1	180.0
K(1)-O(6)#2	2.7011(18)	N(3)-Zn(1)-O(4)#1	89.73(7)
K(1)-O(1)	2.8092(19)	N(3)#1-Zn(1)-O(4)#1	90.27(7)
K(1)-O(5)#3	2.8421(19)	O(3)-Zn(1)-O(4)#1	90.08(7)
K(1)-O(2)	2.8596(17)	O(3)#1-Zn(1)-O(4)#1	89.92(7)
K(1)-O(5)#4	2.8959(19)	N(3)-Zn(1)-O(4)	90.27(7)
K(1)-O(6)#4	2.991(2)	N(3)#1-Zn(1)-O(4)	89.73(7)
K(1)-O(4)#5	3.045(2)	O(3)-Zn(1)-O(4)	89.92(7)
K(1)-O(2)#6	3.0479(19)	O(3)#1-Zn(1)-O(4)	90.08(7)
K(1)-N(8)#4	3.318(2)	O(4)#1-Zn(1)-O(4)	180.0
K(1)-N(5)#3	3.366(2)	O(6)#2-K(1)-O(1)	69.50(6)
K(1)-K(1)#6	4.0405(12)	O(6)#2-K(1)-O(5)#3	145.87(6)
K(1)-K(1)#7	4.4861(12)	O(1)-K(1)-O(5)#3	77.24(6)
K(1)-H(1B)	3.02(3)	O(6)#2-K(1)-O(2)	142.54(6)
N(1)-O(2)	1.239(2)	O(1)-K(1)-O(2)	141.22(6)
N(1)-O(3)	1.267(2)	O(5)#3-K(1)-O(2)	66.21(5)
N(1)-N(2)	1.314(2)	O(6)#2-K(1)-O(5)#4	119.08(6)
N(2)-C(1)	1.366(3)	O(1)-K(1)-O(5)#4	136.97(6)
N(3)-C(1)	1.325(3)	O(5)#3-K(1)-O(5)#4	90.48(5)
N(3)-N(4)	1.360(3)	O(2)-K(1)-O(5)#4	58.48(5)
N(4)-N(5)	1.283(3)	O(6)#2-K(1)-O(6)#4	76.10(5)
N(5)-N(6)	1.354(3)	O(1)-K(1)-O(6)#4	118.70(6)
N(5)-K(1)#8	3.366(2)	O(5)#3-K(1)-O(6)#4	128.92(5)
N(6)-C(1)	1.342(3)	O(2)-K(1)-O(6)#4	95.03(5)
N(6)-N(7)	1.394(2)	O(5)#4-K(1)-O(6)#4	43.24(5)
N(7)-N(8)	1.336(3)	O(6)#2-K(1)-O(4)#5	78.81(5)
N(8)-O(5)	1.237(2)	O(1)-K(1)-O(4)#5	118.58(5)
N(8)-O(6)	1.250(2)	O(5)#3-K(1)-O(4)#5	111.98(5)
N(8)-K(1)#9	3.318(2)	O(2)-K(1)-O(4)#5	67.30(5)
O(1)-H(1A)	0.811(17)	O(5)#4-K(1)-O(4)#5	104.31(5)
O(1)-H(1B)	0.817(18)	O(6)#4-K(1)-O(4)#5	101.66(5)
O(2)-K(1)#6	3.0479(19)	O(6)#2-K(1)-O(2)#6	119.27(5)
O(4)-K(1)#10	3.045(2)	O(1)-K(1)-O(2)#6	75.96(5)
O(4)-H(4B)	0.822(17)	O(5)#3-K(1)-O(2)#6	56.90(5)
O(4)-H(4A)	0.816(18)	O(2)-K(1)-O(2)#6	93.75(5)
O(5)-K(1)#8	2.8420(19)	O(5)#4-K(1)-O(2)#6	63.14(5)
O(5)-K(1)#9	2.8959(19)	O(6)#4-K(1)-O(2)#6	79.13(5)
O(6)-K(1)#11	2.7011(18)	O(4)#5-K(1)-O(2)#6	161.05(5)
O(6)-K(1)#9	2.991(2)	O(6)#2-K(1)-N(8)#4	98.15(5)
		O(1)-K(1)-N(8)#4	127.88(5)
		O(5)#3-K(1)-N(8)#4	108.37(5)
		O(2)-K(1)-N(8)#4	77.96(5)

		O(5)#4-K(1)-N(8)#4	21.62(5)
		O(6)#4-K(1)-N(8)#4	22.07(4)
		O(4)#5-K(1)-N(8)#4	107.19(5)
		O(2)#6-K(1)-N(8)#4	66.81(5)
		O(6)#2-K(1)-N(5)#3	106.21(5)
		O(1)-K(1)-N(5)#3	72.37(5)
		O(5)#3-K(1)-N(5)#3	54.68(5)
		O(2)-K(1)-N(5)#3	76.08(5)
		O(5)#4-K(1)-N(5)#3	131.89(5)
		O(6)#4-K(1)-N(5)#3	168.26(5)
		O(4)#5-K(1)-N(5)#3	68.01(5)
		O(2)#6-K(1)-N(5)#3	108.73(5)
		N(8)#4-K(1)-N(5)#3	153.30(5)
		O(6)#2-K(1)-K(1)#6	158.53(5)
		O(1)-K(1)-K(1)#6	111.63(4)
		O(5)#3-K(1)-K(1)#6	45.78(4)
		O(2)-K(1)-K(1)#6	48.83(4)
		O(5)#4-K(1)-K(1)#6	44.70(4)
		O(6)#4-K(1)-K(1)#6	85.40(4)
		O(4)#5-K(1)-K(1)#6	116.13(4)
		O(2)#6-K(1)-K(1)#6	44.93(3)
		N(8)#4-K(1)-K(1)#6	63.60(4)
		N(5)#3-K(1)-K(1)#6	94.13(4)
		O(6)#2-K(1)-K(1)#7	40.33(4)
		O(1)-K(1)-K(1)#7	96.28(4)
		O(5)#3-K(1)-K(1)#7	156.53(4)
		O(2)-K(1)-K(1)#7	122.43(4)
		O(5)#4-K(1)-K(1)#7	78.87(4)
		O(6)#4-K(1)-K(1)#7	35.76(3)
		O(4)#5-K(1)-K(1)#7	91.03(4)
		O(2)#6-K(1)-K(1)#7	99.71(4)
		N(8)#4-K(1)-K(1)#7	57.83(4)
		N(5)#3-K(1)-K(1)#7	145.17(4)
		K(1)#6-K(1)-K(1)#7	120.45(3)
		O(6)#2-K(1)-H(1B)	59.1(5)
		O(1)-K(1)-H(1B)	15.5(4)
		O(5)#3-K(1)-H(1B)	89.7(5)
		O(2)-K(1)-H(1B)	155.6(4)
		O(5)#4-K(1)-H(1B)	129.0(5)
		O(6)#4-K(1)-H(1B)	103.6(4)
		O(4)#5-K(1)-H(1B)	122.6(6)
		O(2)#6-K(1)-H(1B)	74.8(6)
		N(8)#4-K(1)-H(1B)	115.3(5)

		N(5)#3-K(1)-H(1B)	87.1(4)
		K(1)#6-K(1)-H(1B)	116.6(6)
		K(1)#7-K(1)-H(1B)	81.2(4)
		O(2)-N(1)-O(3)	118.62(18)
		O(2)-N(1)-N(2)	116.71(18)
		O(3)-N(1)-N(2)	124.65(18)
		N(1)-N(2)-C(1)	117.17(17)
		C(1)-N(3)-N(4)	107.04(17)
		C(1)-N(3)-Zn(1)	125.31(15)
		N(4)-N(3)-Zn(1)	127.64(13)
		N(5)-N(4)-N(3)	110.51(17)
		N(4)-N(5)-N(6)	106.37(17)
		N(4)-N(5)-K(1)#8	97.18(13)
		N(6)-N(5)-K(1)#8	107.85(13)
		C(1)-N(6)-N(5)	109.32(17)
		C(1)-N(6)-N(7)	127.29(18)
		N(5)-N(6)-N(7)	122.88(17)
		N(8)-N(7)-N(6)	108.82(16)
		O(5)-N(8)-O(6)	121.6(2)
		O(5)-N(8)-N(7)	123.25(19)
		O(6)-N(8)-N(7)	115.13(19)
		O(5)-N(8)-K(1)#9	59.59(11)
		O(6)-N(8)-K(1)#9	64.08(12)
		N(7)-N(8)-K(1)#9	165.55(14)
		K(1)-O(1)-H(1A)	115.0(19)
		K(1)-O(1)-H(1B)	97(2)
		H(1A)-O(1)-H(1B)	101(3)
		N(1)-O(2)-K(1)	130.67(14)
		N(1)-O(2)-K(1)#6	109.63(13)
		K(1)-O(2)-K(1)#6	86.25(5)
		N(1)-O(3)-Zn(1)	128.81(13)
		Zn(1)-O(4)-K(1)#10	123.75(8)
		Zn(1)-O(4)-H(4B)	112(2)
		K(1)#10-O(4)-H(4B)	100(2)
		Zn(1)-O(4)-H(4A)	116(3)
		K(1)#10-O(4)-H(4A)	92(3)
		H(4B)-O(4)-H(4A)	112(3)
		N(8)-O(5)-K(1)#8	145.65(15)
		N(8)-O(5)-K(1)#9	98.79(13)
		K(1)#8-O(5)-K(1)#9	89.52(5)
		N(8)-O(6)-K(1)#11	162.10(15)
		N(8)-O(6)-K(1)#9	93.85(13)
		K(1)#11-O(6)-K(1)#9	103.90(5)

		N(3)-C(1)-N(6)	106.75(18)
		N(3)-C(1)-N(2)	133.65(19)
		N(6)-C(1)-N(2)	119.54(19)

3. Thermal Behaviors

The thermal behaviors of all target compounds **1~5** were investigated by differential scanning calorimetry (DSC) and thermogravimetric (TG) at a heating rate of 5 °C min^{-1}. The results were shown as follows Figure S6~Figure S10.

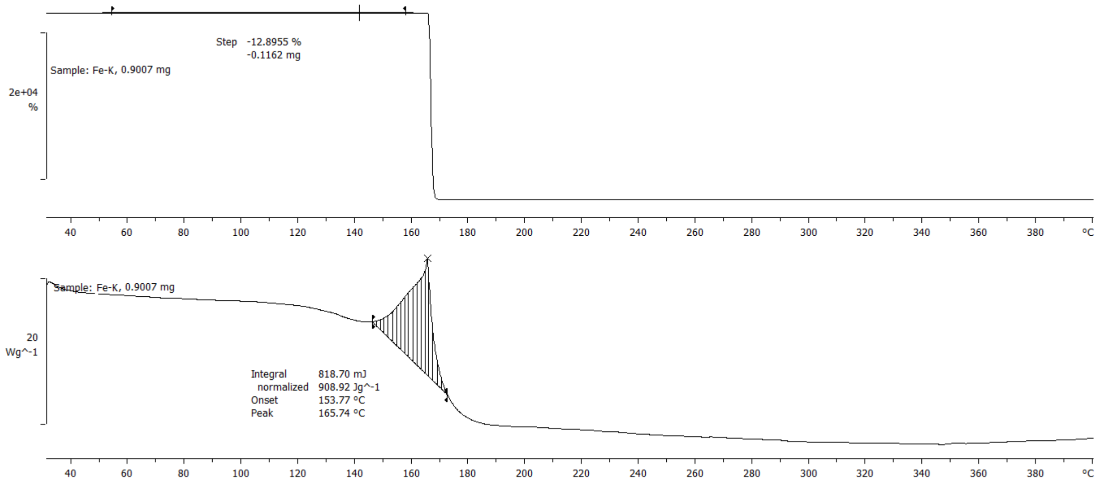

Figure S6. The TG and DSC curves of compound **1** at heating rate of 5 °C min^{-1}

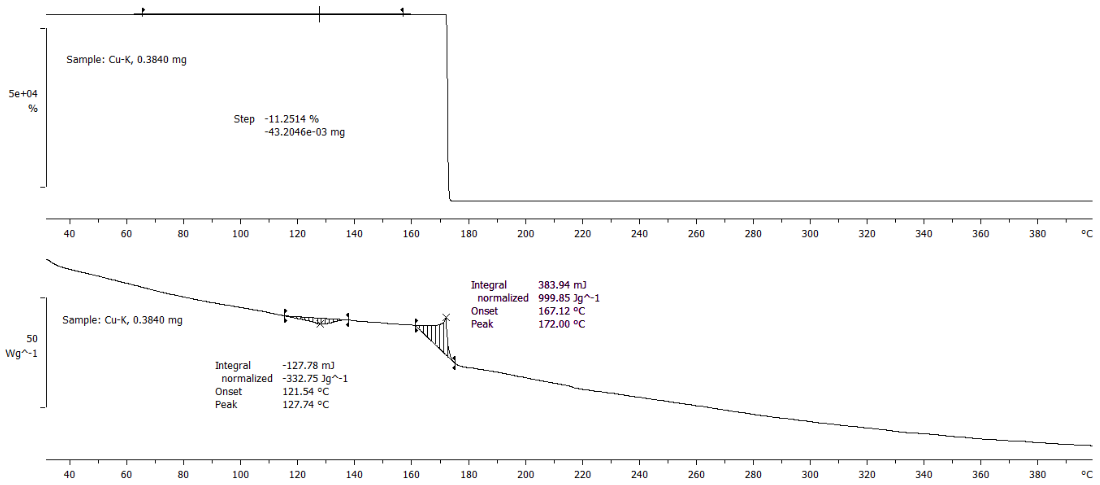

Figure S7. The TG and DSC curves of compound **2** at heating rate of 5 °C min^{-1}

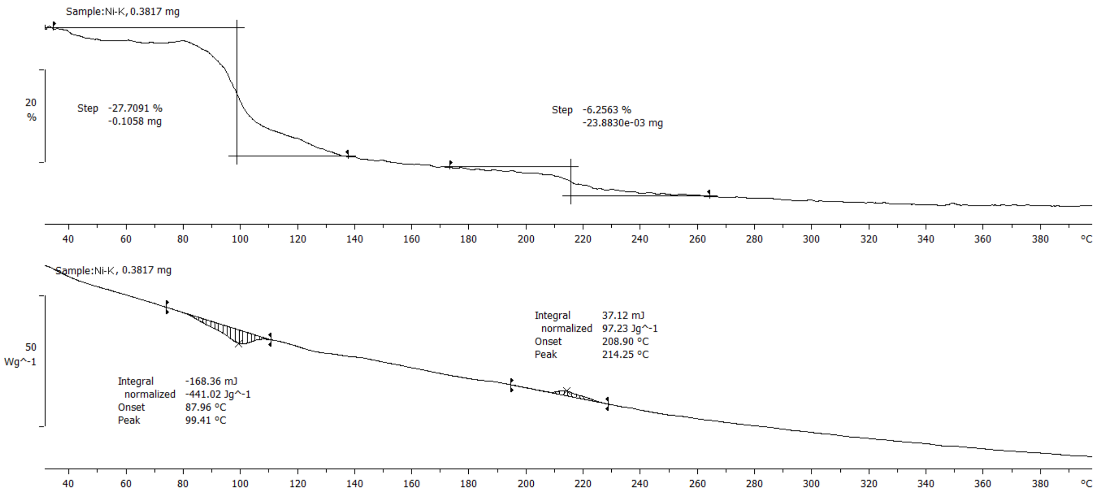

Figure S8. The TG and DSC curves of compound **3** at heating rate of 5 °C min^{-1}

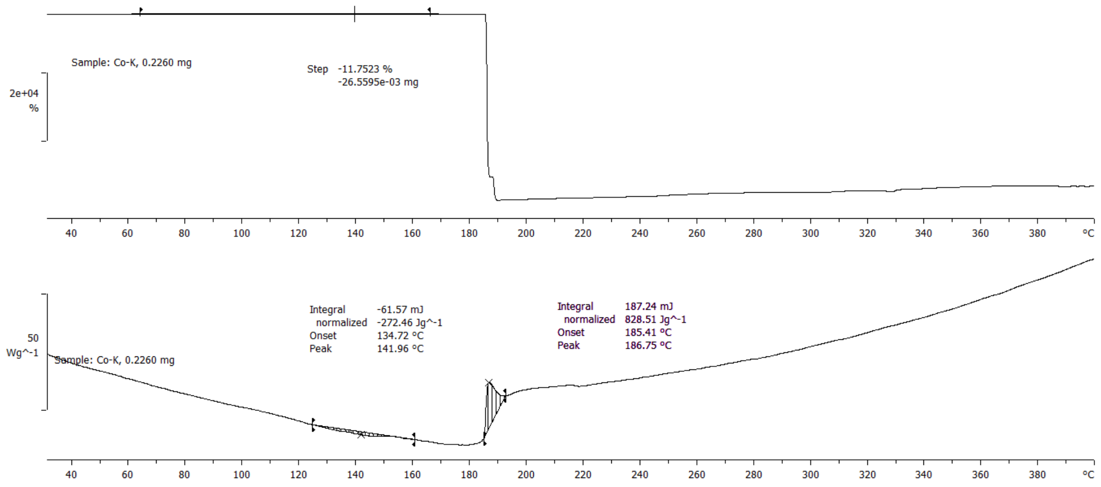

Figure S9. The TG and DSC curves of compound **4** at heating rate of 5 °C min^{-1}

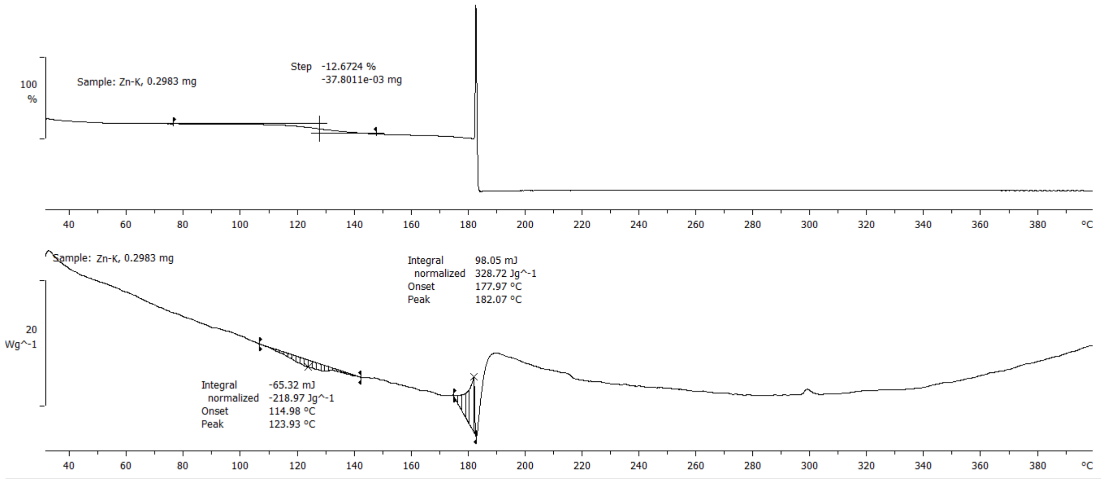

Figure S10. The TG and DSC curves of compound **5** at heating rate of 5 °C min^{-1}

4. Computation Details

The electronic structures of periodic boundary conditions were calculated by DFT method with PBE[5] exchange and correlation and plane wave basis set realized by the CASTEP[6] code. The on the fly generation ultrasoft potential[7] for Materials Studio 8.0 (OTFG_80) and semi-empirical dispersion correction of Tkatchenko and Scheffler[8] were combined with DFT calculations. A cutoff energy of 630 ev and Brillouin zone sampling on a grid of spacing $2\pi\times0.07$ Å^{-1} were set. After testing the total energies of the systems containing transition metal ions, it can be validated that their ground states are high spin states. The experimental and calculated densities of compounds **1~5** are presented as Table S6.

Table S6. The experimental and calculated densities of compounds **1~5**

Compounds	1	2	3	4	5
**Formula**	$C_2H_8N_{16}O_{12}FeK_2$	$C_2H_8N_{16}O_{12}CuK_2$	$C_2H_8N_{16}O_{12}NiK_2$	$C_2H_8N_{16}O_{12}CoK_2$	$C_2H_8N_{16}O_{12}ZnK_2$
**Densities** (Exp., g cm^{-3})	2.095	2.135	2.111	2.124	2.138
**Densities** (Cal., g cm^{-3})	2.123	2.163	2.157	2.178	2.150

The difference between the experimental and calculated densities is very small. It means the computational method is reliable. According to the following chemical equations, the binding energies can be obtained by reaction energies. The below-mentioned isolated states were treated in 20 Å×20 Å×20 Å boxes, respectively. The binding energies of equations 1~5 are showed in Table S7.

$$FeK_2(CN_8O_4)_2(H_2O)_4 \rightarrow 2\times K^+ + Fe^{2+} + 2\times(CN_8O_4)^{2-} + 4\times H_2O \quad (1)$$

$$CuK_2(CN_8O_4)_2(H_2O)_4 \rightarrow 2\times K^+ + Cu^{2+} + 2\times(CN_8O_4)^{2-} + 4\times H_2O \quad (2)$$

$$NiK_2(CN_8O_4)_2(H_2O)_4 \rightarrow 2\times K^+ + Ni^{2+} + 2\times(CN_8O_4)^{2-} + 4\times H_2O \quad (3)$$

$$CoK_2(CN_8O_4)_2(H_2O)_4 \rightarrow 2\times K^+ + Co^{2+} + 2\times(CN_8O_4)^{2-} + 4\times H_2O \quad (4)$$

$$ZnK_2(CN_8O_4)_2(H_2O)_4 \rightarrow 2\times K^+ + Zn^{2+} + 2\times(CN_8O_4)^{2-} + 4\times H_2O \quad (5)$$

Table S7. The binding energies of equations 1~5 / kJ mol^{-1}

Equations	1	2	3	4	5
**Binding Energies**	3654.6	3841.3	3836.4	3847.1	3683.0

As well-known, CBS-QB3[9,10] was realized by Gaussian 09 program[11], which is a high accuracy composite method. The enthalpy of formation of gaseous $CH_2N_8O_4$ was calculated by the atomic reaction shown as below at the CBS-QB3 level is 554.2 kJ mol^{-1}.

$$CH_2N_8O_4 \rightarrow C + 2\times H + 8\times N + 4\times O$$

The enthalpy of formation of gaseous $(CN_8O_4)^{2-}$ was calculated by the protonation shown as below at the CBS-QB3 level is 396.6 kJ mol^{-1}.

$$CH_2N_8O_4 \rightarrow (CN_8O_4)^{2-} + 2\times H^+$$

The enthalpies of formation of solid states can be derived by the binding energies and corresponding enthalpies of formation of isolated states. The enthalpies of formation of metal atoms and metal oxides, and the heats of formation of compounds **1~5** are presented as Table S8~S10, respectively.

Table S8. The enthalpies of formation of metal atoms / kJ mol^{-1}

Metal	K	Fe	Cu	Ni	Co	Zn
**EOF**	89.0	415.5	337.6	430.1	426.7	130.4
**1st IP**	418.8	762.5	745.5	737.1	760.4	906.4
**2nd IP**	—	1561.9	1957.9	1753.0	1648.0	1733.3

Table S9. The enthalpies of formation of metal oxides / kJ mol^{-1}

Compounds	K_2O	FeO	CuO	NiO	CoO	ZnO
**EOF**	-363.2	-272.0	-156.1	-240.0	-237.7	-350.5
Species	H_2O	CO_2	N	O	H	H^+
**EOF**	-241.8	-393.5	472.7	249.2	218.0	1530.0

Table S10. The heats of formation of compounds **1~5** / kJ mol^{-1}

Compounds	**1**	**2**	**3**	**4**	**5**
$\Delta_f H^{\circ}_{(s, M)}$	73.2	-41.3	74.6	170.5	71.3

5. References

[1] (a) Tests were conducted according to the *UN Recommendations on the Transport of Dangerous Goods, Manual of Tests and Criteria*, 5th ed., United Nations Publication, New York, **2009**; (b) 13.4.2 Test 3 (ii) BAM Fallhammer, pp. 75–82; (c) 13.5.1 Test 3 (i): BAM friction apparatus, pp. 104–107.

[2] M. Sucéska, *EXPLO5 v6.04 program*, Brodarski Institute, Zagreb, Croatia, **2017**.

[3] D. Fischer, T. M. Klapötke, J. Stierstorfer, *Angew. Chem. Int. Ed.* **2015**, *54*, 10299–10302.

[4] G. M. Sheldrick, *SHELXS-97, Program for X-ray Crystal Structure Determination*, University of Göttingen, Göttingen, Germany, **1997**.

[5] J. P. Perdew, K. Burke, M. Ernzerhof, *Phys. Rev. Lett.* **1996**, *77*, 3865–3868.

[6] S. J. Clark, M. D. Segall, C. J. Pickard, P. J. Hasnip, M. I. J. Probert, K. Refson, M.C. Payne, *Z. Kristallogr* **2005**, *220*, 567–570.

[7] D. Vanderbilt, *Phys. Rev.* B **1990**, *41*, 7892–7895.

[8] A.Tkatchenko, M. Scheffler, *Phys. Rev. Lett.* **2009**, *102*, 073005.

[9] J. A. Montgomery Jr., M. J. Frisch, J. W. Ochterski, G. A. Petersson, *J. Chem. Phys.* **1999**, *110*, 2822–2827.

[10] J. A. Montgomery Jr., M. J. Frisch, J. W. Ochterski, G. A. Petersson, *J. Chem. Phys.* **2000**, 112, 6532–6542.

[11] M. J. Frisch, G. W. Trucks, H. B. Schlegel, G. E. Scuseria,M. A. Robb, J. R. Cheeseman, J. A. Montgomery Jr, T. Vreven, K. N. Kudin, J. C. Burant, J. M. Millam, S. S. Iyengar, J. Tomasi, V. Barone, B. Mennucci, M. Cossi, G. Scalmani, N. Rega, G. A. Petersson, H. Nakatsuji, M. Hada, M. Ehara, K. Toyota, R. Fukuda, J. Hasegawa, M. Ishida, T. Nakajima, Y. Honda, O.

Kitao, H. Nakai, M. Klene, X. Li, J. E. Knox, H. P. Hratchian, J. B. Cross, V. Bakken, C. Adamo, J. Jaramillo, R. Gomperts, R. E. Stratmann, O. Yazyev, A. J. Austin, R. Cammi, C. Pomelli, J. W. Ochterski, P. Y. Ayala, K. Morokuma, G. A. Voth, P. Sal-vador, J. J. Dannenberg, V. G. Zakrzewski, S. Dapprich, A. D. Daniels, M. C. Strain, O. Farkas, D. K. Malick, A. D. Rabuck, K. Raghavachari, J. B. Foresman, J. V. Ortiz, Q. Cui, A. G. Ba-boul, S. Clifford, J. Cioslowski, B. B. Stefanov, G. Liu, A. Liashenko, P. Piskorz, I. Komaromi, R. L. Martin, D. J. Fox, T. Keith, M. A. Al-Laham, C. Y. Peng, A. Nanayakkara, M. Challacombe, P. M. W. Gill, B. Johnson, W. Chen, M. W. Wong, C. Gonzalez and J. A. Pople, Gaussian 09, rev. B.01, Gaussian, Inc., Wallingford, CT, **2009**.
